# *Plasmodium berghei* MAPK1 Displays Differential and Dynamic Subcellular Localizations during Liver Stage Development

**DOI:** 10.1371/journal.pone.0059755

**Published:** 2013-03-27

**Authors:** Jannika Katharina Wierk, Annette Langbehn, Maria Kamper, Stefanie Richter, Paul-Christian Burda, Volker Theo Heussler, Christina Deschermeier

**Affiliations:** 1 Department of Molecular Parasitology, Bernhard Nocht Institute for Tropical Medicine, Hamburg, Germany; 2 Institute of Cell Biology, University of Bern, Bern, Switzerland; Centre National de la Recherche Scientifique, France

## Abstract

Mitogen-activated protein kinases (MAPKs) regulate key signaling events in eukaryotic cells. In the genomes of protozoan *Plasmodium* parasites, the causative agents of malaria, two genes encoding kinases with significant homology to other eukaryotic MAPKs have been identified (*mapk1, mapk2*). In this work, we show that both genes are transcribed during *Plasmodium berghei* liver stage development, and analyze expression and subcellular localization of the PbMAPK1 protein in liver stage parasites. Live cell imaging of transgenic parasites expressing GFP-tagged PbMAPK1 revealed a nuclear localization of PbMAPK1 in the early schizont stage mediated by nuclear localization signals in the C-terminal domain. In contrast, a distinct localization of PbMAPK1 in comma/ring-shaped structures in proximity to the parasite’s nuclei and the invaginating parasite membrane was observed during the cytomere stage of parasite development as well as in immature blood stage schizonts. The PbMAPK1 localization was found to be independent of integrity of a motif putatively involved in ATP binding, integrity of the putative activation motif and the presence of a predicted coiled-coil domain in the C-terminal domain. Although PbMAPK1 knock out parasites showed normal liver stage development, the kinase may still fulfill a dual function in both schizogony and merogony of liver stage parasites regulated by its dynamic and stage-dependent subcellular localization.

## Introduction

Protozoan parasites of the genus *Plasmodium,* the causative agents of malaria, proliferate in a complex life cycle, comprising both asexual replication in the liver and the blood of the mammalian host organism and sexual reproduction followed by asexual replication in the disease-transmitting *Anopheles* mosquito vector. The asymptomatic liver stage development of the parasite is initiated by the invasion of a host hepatocyte by a single *Plasmodium* sporozoite and results in the rapid production of several thousands of infectious merozoites that are released in the bloodstream, initiating the symptomatic phase of the disease. During liver stage development, the parasite resides inside its host cell surrounded by two membranes: the parasite plasma membrane (PPM) and the parasitophorous vacuole membrane (PVM). The parasite develops initially by extensive nuclear division without cytokinesis to form a multinuclear syncytium, the schizont. Then, by invagination of the PPM, single merozoites are formed which are still confined within the PVM. Only after PVM breakdown, parasite-filled vesicles (merosomes) bud off the infected cell into the liver sinusoids [1]. Via the bloodstream, unrecognized by the host immune system, the merosomes reach the lung microvasculature where the infectious merozoites are released [2].

Passing through this life cycle, *Plasmodium* parasites are subject to drastic environmental changes: they shuttle between extra- and intracellular stages and efficiently proliferate both in highly specialized, metabolically limited red blood cells and in hepatocytes. In the latter, metabolically active cells, *Plasmodium* parasites multiply at a tremendous rate, generating several thousands of merozoites in just a few days. Although nuclear division and subsequent organelle distribution during blood and liver stage schizogony/merogony have recently been described morphologically [3], [4], the intracellular signaling events underlying these processes and their rapid and reliable performance are still largely unknown. However, it was reasoned that protein kinases may play a role [3], [5].

In eukaryotic cell signal transduction, both extra- and intracellular mitotic and stress-related stimuli can result in the activation of serine/threonine protein kinases of the mitogen-activated protein kinase (MAPK) family. While in mammalian cells a whole system of MAPKs belonging to different subfamilies has been described along with their respective upstream kinases, downstream targets and scaffold proteins [6], [7], much less is known about MAPKs in other eukaryotic organisms. In *Plasmodium falciparum*, sequence analyses revealed two MAPK orthologs designated as PfMAPK1 [8], [9] and PfMAPK2 [10]. Subsequent phylogenetic analysis showed that these two sequences do indeed cluster with the MAPK family [11]. Using reverse genetics approaches MAPK1 was shown to be dispensable during blood and mosquito stage development in both *Plasmodium falciparum* [12] and the rodent malaria parasite *Plasmodium berghei* [5]. While MAPK2 was found to be dispensable in asexual blood stages but essential for male gametogenesis in *P. berghei* [13], [14], [15], a vital function of this kinase in asexual blood stage parasites was demonstrated in *P. falciparum* [12].

Structurally, both kinases consist of a kinase domain followed by a C-terminal extension; MAPK2 also has a short N-terminal extension. The kinase domains show clear homology to members of the mammalian MAPK family and harbor protein kinase-specific signature motifs such as the universally conserved DFG motif involved in ATP positioning [16] and, in the case of MAPK1, the MAPK-specific TXY activation motif [6]. Strikingly, the C-terminal domain of MAPK1, but not of MAPK2, differs fundamentally in length and amino acid sequence between human and rodent malaria parasites.

In this work, we analyze for the first time the expression and subcellular localization of PbMAPK1 during *P. berghei* liver stage development, the phase of the parasite’s life cycle where both nuclear division and membrane dynamics have to occur at a dramatic rate, but nevertheless with high accuracy. The localization of the kinase during liver stage development is investigated by live imaging of transgenic parasites expressing GFP-tagged PbMAPK1-fusion proteins and structural determinants regulating subcellular localization of PbMAPK1 are characterized.

## Results

### Expression and Subcellular Localization of Endogenous PbMAPK1 in *P. berghei* Liver Stage Parasites

**Figure 1:**
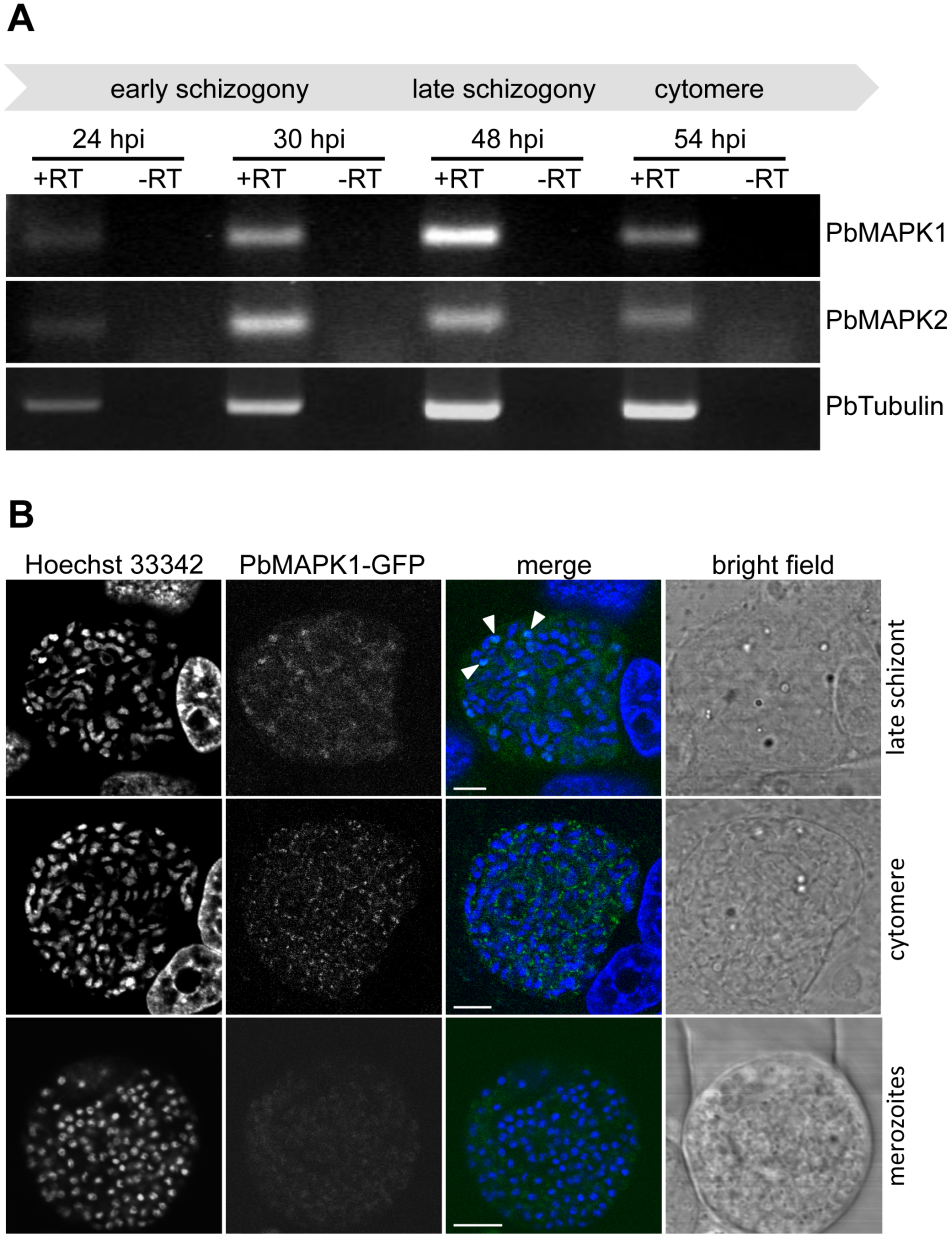
Expression and localization of *P. berghei* MAPK1 during liver stage development. (**A**) **Transcripts encoding PbMAPK1 and PbMAPK2 are detectable in** ***P. berghei*** **liver stage parasites.** Total RNA was prepared from *P. berghei*-infected HepG2 cells 24 hpi, 30 hpi, 48 hpi and 54 hpi. RT-PCR analysis was performed using primer pairs specific for *pbmapk1*, *pbmapk2*, and the constitutively expressed *pbtubulin*. To rule out false positive results originating from gDNA contamination, samples lacking reverse transcriptase (-RT) were processed in parallel. (**B**) **Subcellular localization of the endogenous PbMAPK1 protein during liver stage development.** HepG2 cells were infected with *Pb* ^end^PbMAPK1-GFP parasites in which the endogenous *mapk1* locus has been *gfp*-tagged by homologous recombination. Expression and localization of the PbMAPK1-GFP fusion protein was assayed at different time points of liver stage development (late schizont, cytomere, merozoites) by confocal live cell imaging. Parasite and host cell nuclei were visualized using Hoechst 33342. Arrowheads indicate partial co-localization of PbMAPK1 with parasite nuclei in the late schizont. Scale bars: 5 µm.

RT-PCR analysis revealed transcription of the *pbmapk1* and *pbmapk2* genes in *P. berghei* liver stage parasites (**Figure 1A**). In order to analyze expression and localization of the PbMAPK1 protein, we modified the genomic *pbmapk1* locus by introduction of the GFP ORF sequence downstream of and in frame with the PbMAPK1 coding sequence (**Figure S1A**). The resulting recombinant parasites (*Pb* ^end^PbMAPK1-GFP) express a PbMAPK1-GFP fusion protein under the control of the endogenous PbMAPK1 promoter. Correct integration of the plasmid construct by single cross-over was confirmed by PCR analysis (**Figure S1B**); expression of the 97 kDa full-length PbMAPK1-GFP fusion protein was shown by western blot analysis of mixed blood stage parasites (**Figure S1C**). Live cell imaging of mouse erythrocytes infected with *Pb* ^end^PbMAPK1-GFP parasites revealed a very weak fluorescence showing a non-homogeneous distribution in immature schizonts (**Figure S1D**).

To study PbMAPK1 expression and localization in *P. berghei* liver stage parasites, HepG2 cells were infected with *Pb* ^end^PbMAPK1-GFP sporozoites. At early stages of parasite development, no GFP fluorescence was detectable using live cell imaging techniques. Nevertheless, the presence of very low amounts of the protein could be proven by immunofluorescence analysis of infected cells fixed at 32 hpi, using an anti-GFP antibody (data not shown). At later stages of parasite development (late schizogony, cytomere stage, merozoite formation), live cell imaging was technically challenging but revealed a partially nuclear/partially cytosolic distribution in late schizonts, a non-homogenous distribution of the fusion protein in comma-shaped structures in the proximity of the parasite nuclei at the cytomere stage and a homogenous cytosolic localization in merozoites (**Figure 1B**).

Thus, the PbMAPK1-GFP fusion protein expressed under the control of the endogenous *pbmapk1* promoter is detectable throughout liver stage development and shows a stage-dependent localization pattern.

### Detailed Analysis of Stage-specific Subcellular Localization of PbMAPK1 in *P. berghei* Liver Stage Parasites

**Figure 2:**
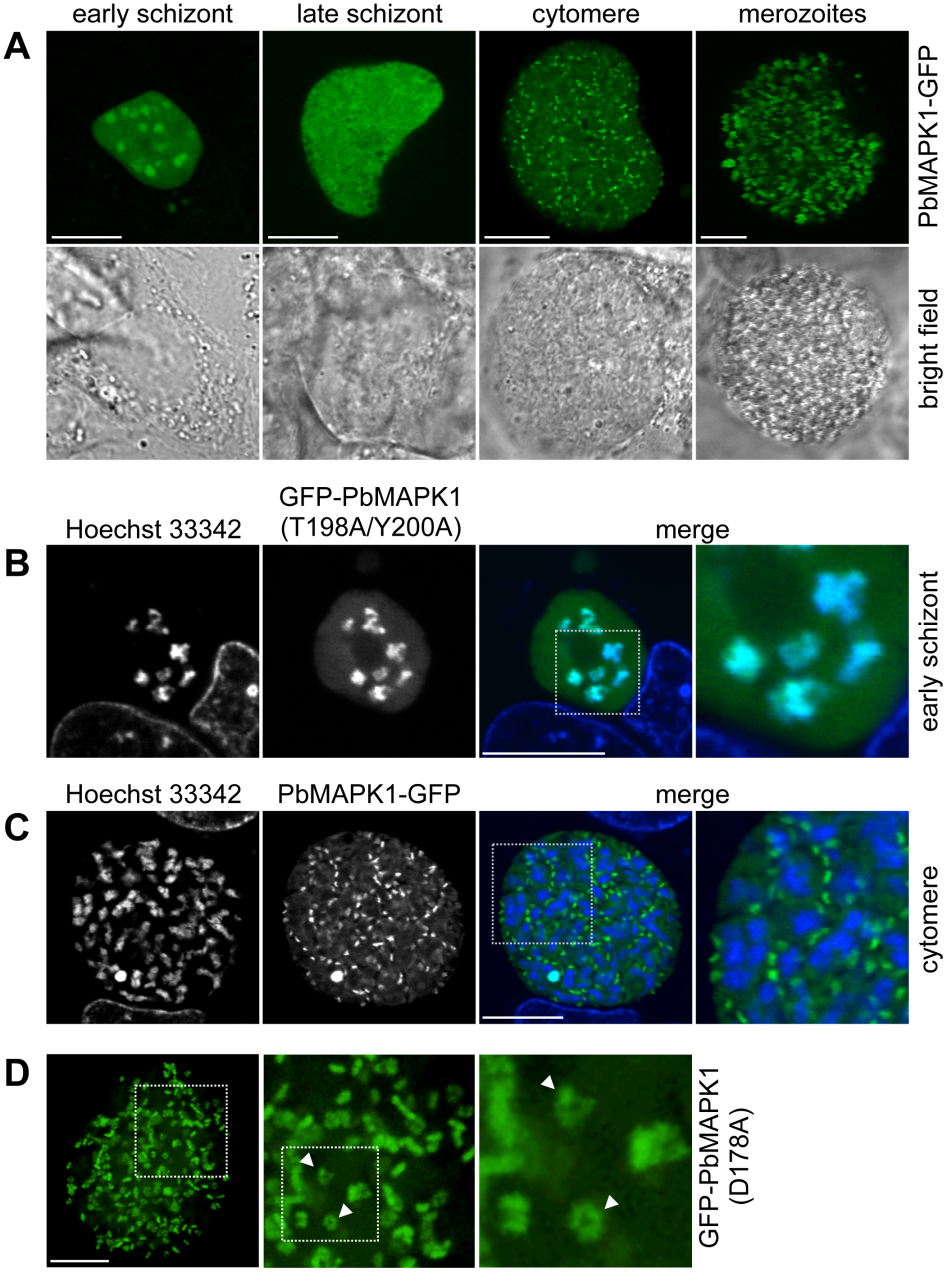
PbMAPK1 displays differential and dynamic subcellular localization during liver stage development. (**A**) **Time course of PbMAPK1 localization (live cell imaging). HepG2 cells were infected with** ***Pb*** **^LS^PbMAPK1-GFP parasites.** Live cell imaging was performed at different developmental stages (early schizont ≈30 hpi, late schizont ≈48 hpi, cytomere ≈54 hpi, merozoites ≈60 hpi). (**B**)**,** (**C**) **Co-staining with nuclei at early schizont and cytomere stages (live cell imaging).** HepG2 cells were infected with *P. berghei* parasites expressing GFP-tagged PbMAPK1. 30 hpi (B, early schizont, *Pb* ^con^GFP-PbMAPK1(T198A/Y200A)) and 54 hpi (C, cytomere, *Pb* ^LS^PbMAPK1-GFP), cells were stained with Hoechst 33342 to visualize host cell and parasite nuclei. Areas containing details additionally displayed at a higher magnification are highlighted in the merged pictures. (**D**) Spinning disc microscopy (live cell imaging). HepG2 cells infected with *Pb* ^con^GFP-PbMAPK1(D178A) parasites were analyzed by spinning disc live microscopy at 54 hpi (cytomere stage). Highlighted area is also shown at higher magnification; arrowheads indicate ring shaped structures. Scale bars: 10 µm.

**Figure 3:**
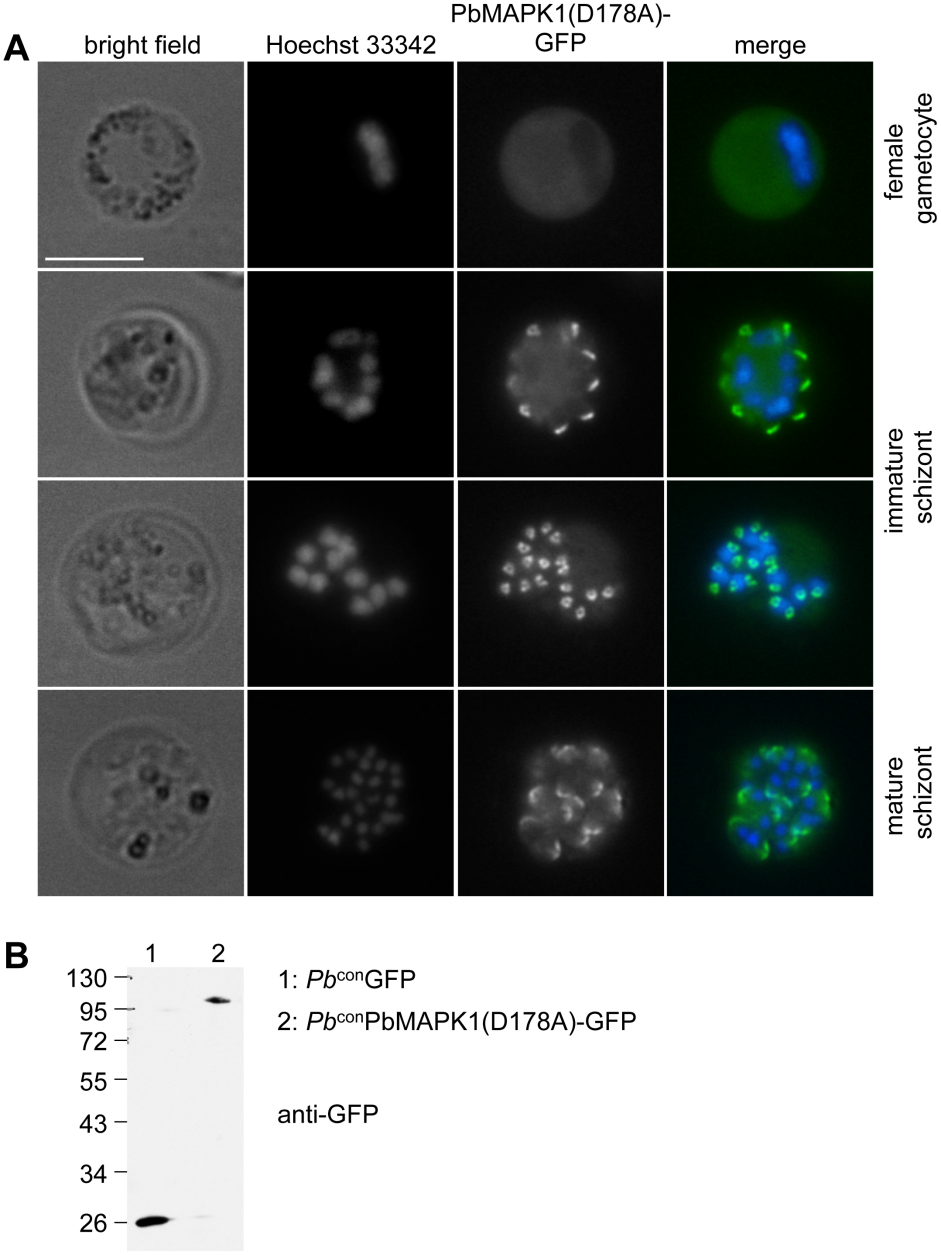
PbMAPK1 displays a distinct localization in *P. berghei* blood stage schizonts. (**A**) *Pb* ^con^PbMAPK1(D178A)-GFP parasites originating from infected mouse blood were analyzed by live epifluorescence microscopy. Parasite nuclei were visualized using Hoechst 33342. Scale bar: 10 µm. (**B**) Western blot analysis of *Pb* ^con^GFP and *Pb* ^con^PbMAPK1(D178A)-GFP parasites. Mixed blood stage parasite protein extracts were prepared from *P. berghei* infected mouse blood, separated by SDS-PAGE and blotted onto nitrocellulose. Detection was performed using mouse anti-GFP/anti-mouse HRP. Molecular weight of marker proteins: kDa; expected molecular weights: GFP: 26 kDa, PbMAPK1(D178A)-GFP: 97 kDa.

To overcome the technical limitations with live cell imaging caused by the low expression level of the PbMAPK1-GFP fusion protein under the control of the endogenous *pbmapk1* promoter, we generated several plasmid constructs suitable for overexpression of GFP-tagged PbMAPK1 and mutants thereof in *P. berghei* blood and liver stage parasites (**Table S1**). Confocal live cell imaging of parasites expressing PbMAPK1-GFP revealed a dynamic, stage-specific localization of the respective fusion protein (**Figure 2A**). While the PbMAPK1 protein was found to be enriched in the parasite nuclei at the early schizont stage (**Figure 2B**), a uniform cytosolic localization was observed at late schizont stages. This rapidly changed during the cytomere stage to transient, comma-shaped structures that did not co-localize with parasite nuclei (**Figure 2C**) and then back to a uniform cytosolic distribution in mature merozoites (**Movie S1**). Quantification revealed a ratio of 1.0+/−0.2 comma-shaped structures per parasite nucleus (n = 8 parasites). Detailed analysis of the comma-shaped structures by spinning disc microscopy followed by 3D reconstruction showed at least some of them to be projections of three-dimensional ring-shaped structures (**Figure 2D**, ****Movie S2****). Similar structures were also observed in immature blood stage schizonts, but not in other blood stage parasites such as female gametocytes (**Figure 3A**). The observed time course of PbMAPK1 subcellular localization was found to be independent of the integrity of the DFG motif putatively involved in ATP positioning (and thus kinase activity) (PbMAPK1(D178A)), integrity of the putative activation motif (PbMAPK1(T198A/Y200A)) and the presence of a putative coiled-coil domain in the kinase’s C-terminal domain (PbMAPK1-Δcc) (**Figure 2** **and** **Figure S2**). The observed localizations were identical for parasite lines expressing PbMAPK1 N-terminally and C-terminally fused to GFP and independent of the promoter (constitutive EEF1alpha promoter or liver stage-specific promoter [17]) used to control the expression of the fusion protein (**Figure 2**). Expression of the full-length PbMAPK1-GFP fusion protein was confirmed by western blot analysis of mixed blood stage parasites (**Figure 3B**).

In *P. falciparum* blood stage parasites, western blot analyses indicated proteolytic processing of the kinase [18]. For liver stage parasites, western blot analyses are technically difficult due to the naturally low infection rates and the resulting tremendous excess of host cell proteins present in the samples. To nonetheless address the question of MAPK1 processing in liver stage parasites, an mCherry-PbMAPK1-GFP fusion protein was expressed in *P. berghei* liver stage parasites; an incomplete co-localization of red and green signals in live cell imaging experiments would hint towards a proteolytic processing of the kinase. However, no proteolytic processing of PbMAPK1 in liver stage parasites was evident using this approach (**Figure S3**).

### Nuclear Localization of PbMAPK1 is Mediated by Nuclear Localization Signals (NLSs) in the C-terminal Domain

**Figure 4:**
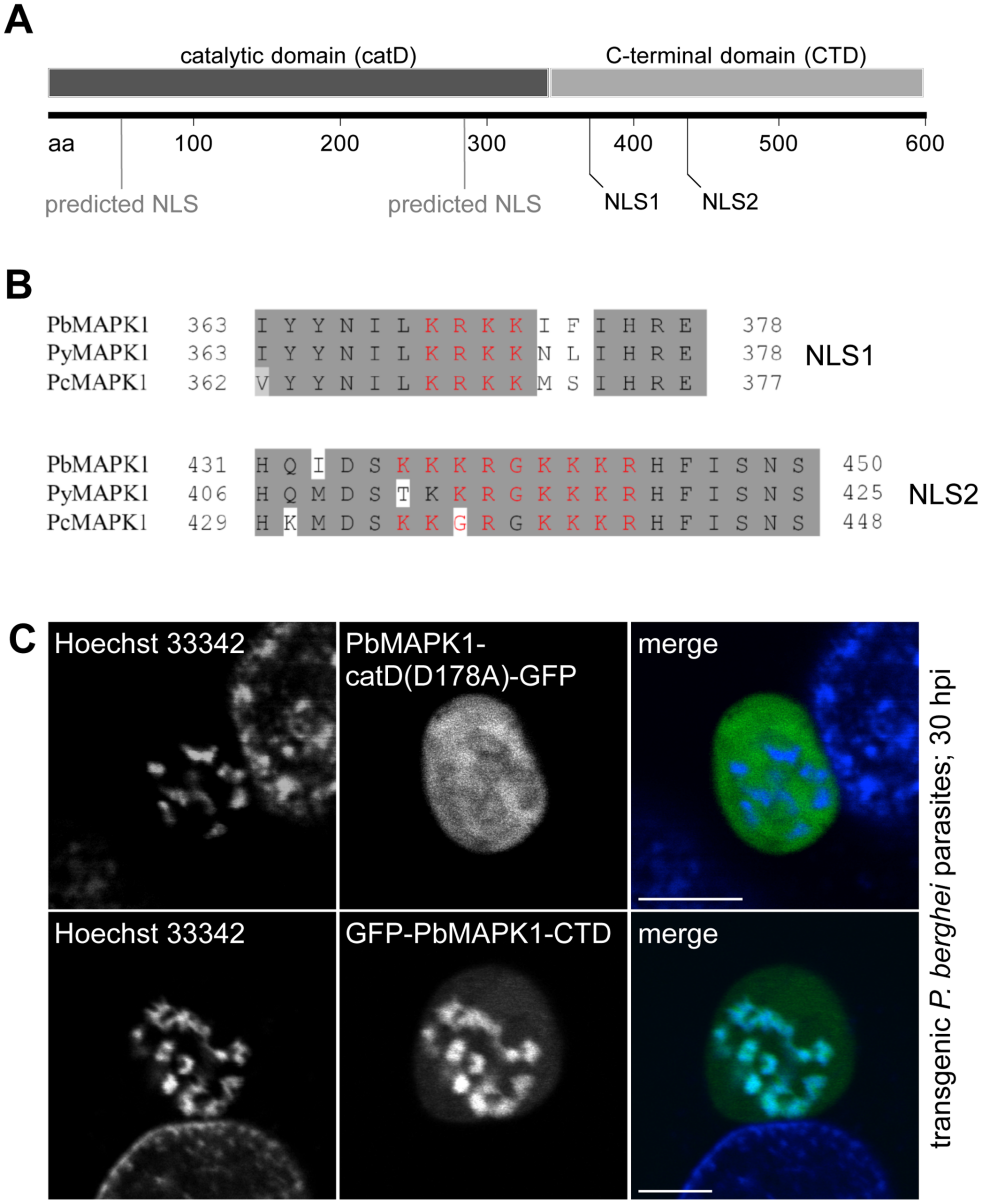
Nuclear localization of PbMAPK1 is mediated by functional NLSs in the C-terminal domain. (**A**) Schematic representation of PbMAPK1 domain structure and position of predicted nuclear localization signals in the catalytic domain (aa 41–57; aa 285–289) and the C-terminal domain (aa 369–372; aa 436–444); aa: amino acids. (**B**) Alignment of putative nuclear localization signals NLS1 and NLS2 (red) in the PbMAPK1 C-terminal domains of rodent malaria parasites *P. berghei* (Pb), *P. yoelii* (Py) and *P. chabaudi* (Pc). (**C**) HepG2 cells were infected with either *Pb* ^con^PbMAPK1-catD(D178A)-GFP or *Pb* ^con^GFP-PbMAPK1-CTD parasites. 30 hpi host cell and parasite nuclei were stained using Hoechst 33342 and live cell imaging was performed. Scale bar: 5 µm.

Primary structure analysis of MAPK1 in different rodent malaria species revealed four sequence segments with putative NLS function, two of them residing in the catalytic and the C-terminal domain, respectively (**Figure 4A, B**). To test the functionality of these putative NLSs in *P. berghei* liver stage parasites, HepG2 cells infected with transgenic parasites expressing GFP fused to either the catalytic domain (PbMAPK1-catD(D178A)-GFP) or to the C-terminal domain (GFP-PbMAPK1-CTD) of PbMAPK1 were analyzed 30 hpi by confocal live cell imaging. While the 68 kDa PbMAPK1-catD(D178A)-GFP fusion protein was found in the parasite cytosol excluded from the parasite nuclei, the GFP-PbMAPK1-CTD fusion protein showed a clear nuclear localization (**Figure 4C**). Because the calculated molecular weights of 97 kDa for the GFP-tagged PbMAPK1 full-length protein and of 59 kDa for GFP-PbMAPK1-CTD both exceed the nuclear pore size exclusion limit of approximately 40–50 kDa [**19**], this is indicative of an active transport of those fusion proteins into the nucleus. Thus, the C-terminal domain is necessary and sufficient to target the kinase to the parasite nuclei.

### Characterization of PbMAPK1 NLSs in HepG2 Cells and *P. berghei* Liver Stage Parasites

**Figure 5:**
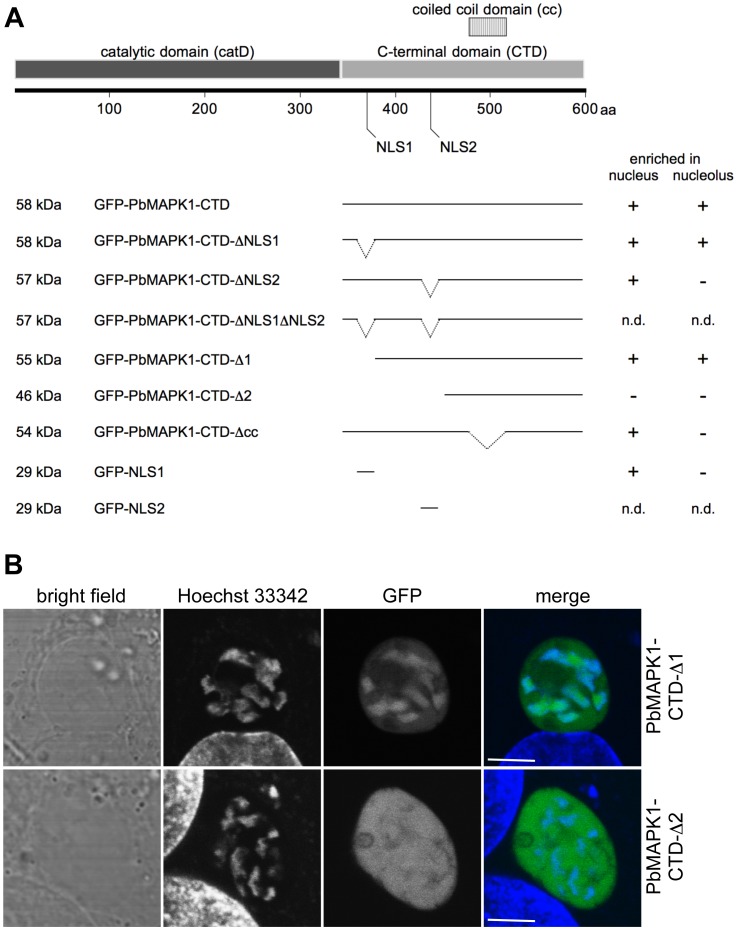
Characterization of PbMAPK1 NLSs in HepG2 cells and *P. berghei* liver stage parasites. (**A**) Schematic representation of PbMAPK1-CTD mutants tested in HepG2 cells and summary of results (for confocal images see Figure S4). HepG2 cells were transiently transfected with pEGFP-C2-based plasmids encoding the indicated GFP-PbMAPK1-CTD deletion constructs. 24 hours post transfection, nuclei were stained using Hoechst 33342 and subcellular localization of the fusion proteins was analyzed by live cell imaging; aa: amino acids; n.d.: not determined (due to very low expression levels of the respective fusion proteins). (**B**) HepG2 cells were infected with either *Pb* ^con^GFP-PbMAPK1-CTD-Δ1 or *Pb* ^con^GFP-PbMAPK1-CTD-Δ2 parasites. 32 hpi host cell and parasite nuclei were stained using Hoechst 33342 and live cell imaging was performed. Scale bar: 5 µm.

To analyze the functionality of the NLSs localized in the PbMAPK1 C-terminal domain in more detail, we generated a variety of different deletion mutants. Due to the fact that the nuclear import/export machinery is at least partially conserved between mammalian cells and apicomplexan parasites [19] and translocation of PfMAPK1 to the nucleus of mammalian cells has been previously reported [18], we decided to use HepG2 cells transiently transfected with pEGFP-C2-based plasmids encoding either GFP-PbMAPK1-CTD or mutants thereof as a model system (see **Figure 5A** for schematic representation of mutants and summary of results and **Figure S4** for confocal microscopy images). In comparison with the procedures for the generation of transgenic *P. berghei* parasite lines, this approach saves time and, importantly, large numbers of laboratory animals.

While GFP alone (encoded by the empty pEGFP-C2 vector) could be observed evenly distributed between the nucleus and the cytoplasm (due to its molecular weight being well below the nuclear pore size exclusion limit), the GFP-PbMAPK1-CTD fusion protein clearly localized to the nuclei of transfected cells (**Figure S4**). There, the protein was specifically enriched at the nucleoli as has been proven by co-localization of the GFP-signal with the nucleolar marker protein fibrillarin (**Figure S5**). Deletion mutants lacking either NLS1 (GFP-PbMAPK1-CTD-ΔNLS1, GFP-PbMAPK1-CTD-Δ1) or NLS2 (GFP-PbMAPK1-CTD-ΔNLS2) still localized to the nucleus while a 46 kDa construct missing both NLSs (GFP-PbMAPK1-CTD-Δ2) was found evenly distributed between the nucleus and the cytoplasm. Correspondingly, a fusion protein consisting of GFP and the isolated NLS1 (GFP-NLS1) was readily transported to the nucleus (the analogous GFP-NLS2 protein was apparently unstable in HepG2 cells and could not be detected by live cell fluorescence microscopy as was also the case for GFP-PbMAPK1-CTD-ΔNLS1ΔNLS2). Thus, the presence of one of the NLSs is necessary and sufficient to promote nuclear import. Additionally, deletion of NLS2 (GFP-PbMAPK1-CTD-ΔNLS2) or the putative coiled-coil domain (GFP-PbMAPK1-CTD-Δcc) abolished nucleolar enrichment of the respective fusion proteins in HepG2 cells.

To confirm that transfected HepG2 cells are a suitable model system, the subcellular localization of two of the fusion proteins was assayed in *P. berghei* liver stage parasites. As in the transfected HepG2 cells, the fusion protein GFP-PbMAPK1-CTD-Δ1 co-localized with the nuclei while GFP-PbMAPK1-CTD-Δ2 was found evenly distributed between cytosol and nuclei (**Figure 5B**).

### The Developmentally Regulated Re-localization of PbMAPK1 from the Nucleus to Extra-nuclear Comma/Ring-shaped Structures is Dependent on both the Catalytic and the C-terminal Domain

**Figure 6:**
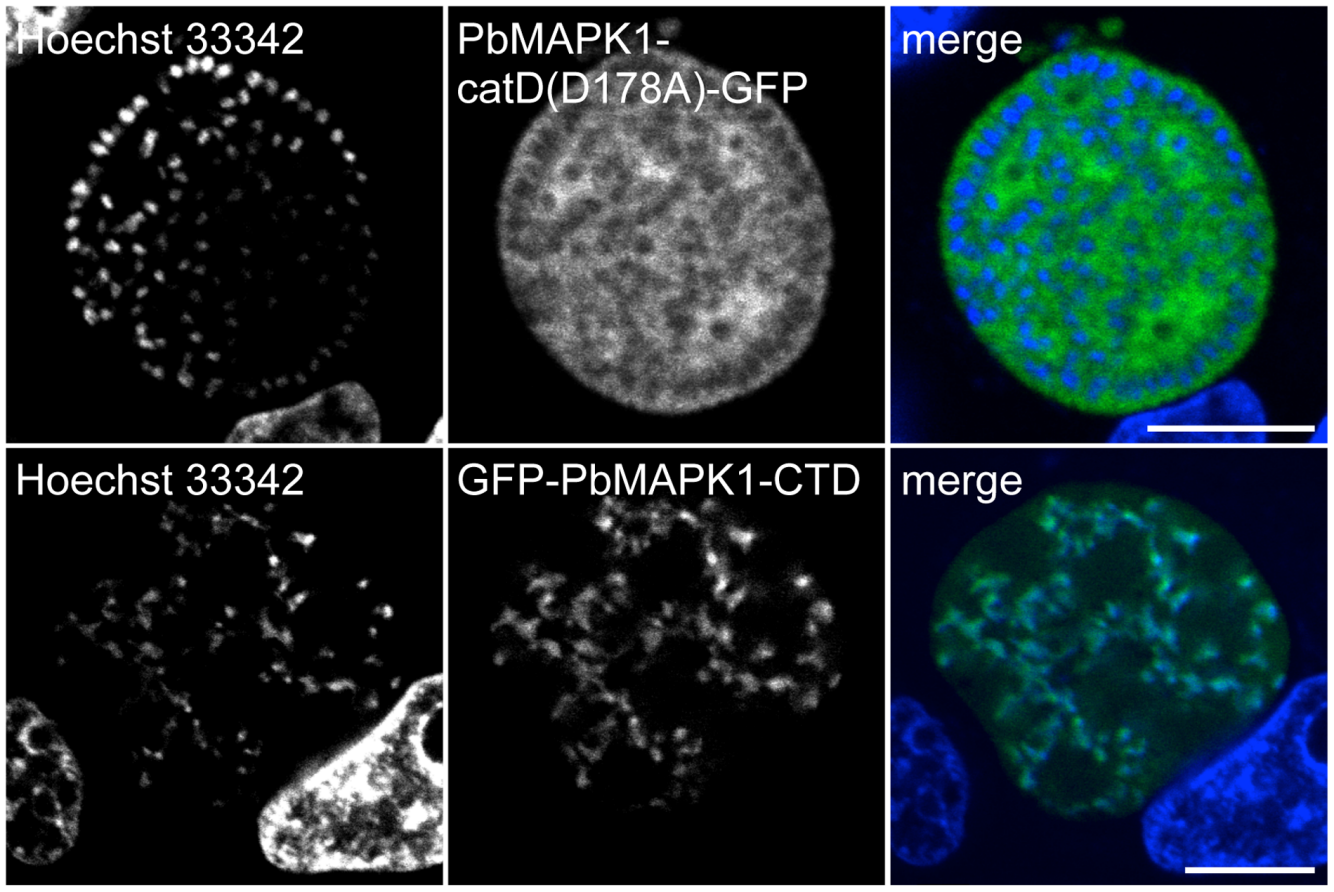
The developmentally regulated re-localization of PbMAPK1 from the nucleus to extra-nuclear comma/ring-shaped structures is dependent on both the catalytic and the C-terminal domain. HepG2 cells were infected with either *Pb* ^con^PbMAPK1-catD(D178A)-GFP or *Pb* ^con^GFP-PbMAPK1-CTD parasites. 54 hpi host cell and parasite nuclei were stained using Hoechst 33342 and live cell imaging was performed. Scale bar: 10 µm.

To investigate if the localization of PbMAPK1 to comma/ring-shaped extra-nuclear structures at the cytomere stage is determined by either the catalytic domain or the C-terminal domain of the kinase alone, HepG2 cells were infected with transgenic parasites expressing GFP fused to either the catalytic domain (*Pb* ^con^PbMAPK1-catD(D178A)-GFP) or the C-terminal domain (*Pb* ^con^GFP-PbMAPK1-CTD) of PbMAPK1. As at 30 hpi (**Figure 4**), live cell imaging of cytomere stage parasites at 54 hpi revealed a cytosolic, extra-nuclear distribution of the PbMAPK1-catD(D178A)-GFP fusion protein while the GFP-PbMAPK1-CTD fusion protein still co-localized with the parasite nuclei (**Figure 6**). Thus, nuclear export of full-length PbMAPK1 after early schizont stage depends on the presence of the catalytic domain. On the other hand, the catalytic domain alone is not sufficient to induce localization of a GFP fusion protein to the comma/ring-shaped structures observed for the full-length protein at the cytomere stage. This indicates a role for the C-terminal domain in targeting the full-length kinase to these structures once the kinase is no longer in the nucleus.

### PbMAPK1 Knockout Parasites Show Normal Liver Stage Development

As has been shown previously, *P. berghei* parasites lacking a functional MAPK1 enzyme develop normally during blood and mosquito stages [5]. To analyze liver stage development of these parasites, HepG2 cells were infected with PbMAPK1 knockout parasites (*map-1* mutant; kind gift of R. Tewari). No significant differences to wildtype parasites were found neither in the ability of the PbMAPK1 knockout sporozoites to invade host cells nor in their successful completion of *in vitro* liver stage development by the formation of detached cells (**Figure S6A, B**). Immunofluorescence analysis of parasites at 54 hpi revealed phenotypically normal initiation and progression of the cytomere stage and merozoite formation (**Figure S6C**).

As the *pbmapk2* transcript is present in both *P. berghei* wildtype (**Figure 1A**) and PbMAPK1 knockout liver stage parasites (data not shown) the PbMAPK2 protein may be expressed and may compensate for the loss of PbMAPK1 in the knockout parasites. Indeed, Dorin-Semblat *et al.* observed an upregulation of the PfMAPK2 protein in *pfmap-1* knockout parasites [12]. To evaluate if mutually redundant functions of PbMAPK1 and PbMAPK2 may exist, we analyzed the stage-specific subcellular localization of PbMAPK2 by infecting HepG2 cells with transgenic *P. berghei* parasites expressing GFP-tagged PbMAPK2 under the control of the constitutive EEF1alpha promoter. The fusion protein was found both within and in close proximity to the parasite nuclei throughout liver stage development. In addition, a localization of the kinase between the dividing nuclei was observed during both the early and late schizont stage (i.e. during karyokinesis) (**Figure S7**). Thus, the two kinases display different localization patterns, especially during late liver stage development, calling into question the hypothesis that PbMAPK2 may compensate for the loss of PbMAPK1.

## Discussion

In *Plasmodium* parasites, two MAPK isoforms have been described, designated as MAPK1 and MAPK2. While both the MAPK1 transcript and the MAPK1 protein were detected in asexual blood stage parasites by northern and western blot analysis [8], [18], the MAPK2 gene was found to be expressed mainly in gametocytes [10], [14], although a low abundance of the protein in *P. falciparum* asexual blood stage parasites has been shown [12]. Furthermore, Nivez et al. [20] detected transcription of the MAPK1 gene in *P. yoelii* liver stage parasites. In this work, we showed that transcripts encoding both PbMAPK1 and PbMAPK2 were detectable in *P. berghei* liver stage parasites and confirmed the presence of the PbMAPK1 protein throughout liver stage development by *gfp*-tagging of the endogenous *pbmapk1* gene.

Live cell imaging of *P. berghei* parasites expressing GFP-tagged MAPK1 under the control of the endogenous *pbmapk1* promoter revealed a stage-dependent localization pattern with a partially nuclear localization of the kinase during schizogony followed by a comma-shaped pattern in the vicinity of the parasite nuclei during cytomere stage. Nevertheless, expression of the fusion protein was low, resulting in a very weak GFP-fluorescence not allowing live cell imaging of early liver stage parasites and preventing the detailed analysis of the localization pattern in late liver stage parasites. Thus, we decided to express the PbMAPK1-GFP fusion protein under the control of a stronger, either constitutive (EEF1a) or liver stage-specific promoter. In addition, a construct allowing the expression of a GFP-PbMAPK2 fusion protein under the control of the constitutive EEF1a promoter was generated.

In live cell imaging experiments, the GFP-tagged PbMAPK2 protein was found in, around and between the dividing parasite nuclei during schizogony, with a localization pattern resembling the distribution of α-tubulin observed in *P. falciparum* blood stage schizonts [3], [21], suggesting a function of this kinase in liver stage parasite karyokinesis. Correspondingly, detailed analysis of the defective male gametocyte development of MAPK2 knockout parasites revealed normal DNA synthesis but a failure in subsequent karyokinesis/cytokinesis [12], [14], [15], [22]. Thus, MAPK2 may be involved in cell division processes mainly in phases of life cycle with rapid nuclear division (liver stage, male gametocytogenesis), a hypothesis that is further supported by the finding that PfMAPK2 can be phosphorylated/activated *in vitro* by PfNEK-1, an enzyme closely related to the never-in-mitosis/Aspergillus (NIMA)/NIMA-like kinase (NEK) family of protein kinases whose members have been found to be involved in cell cycle control [23].

**Figure 7:**
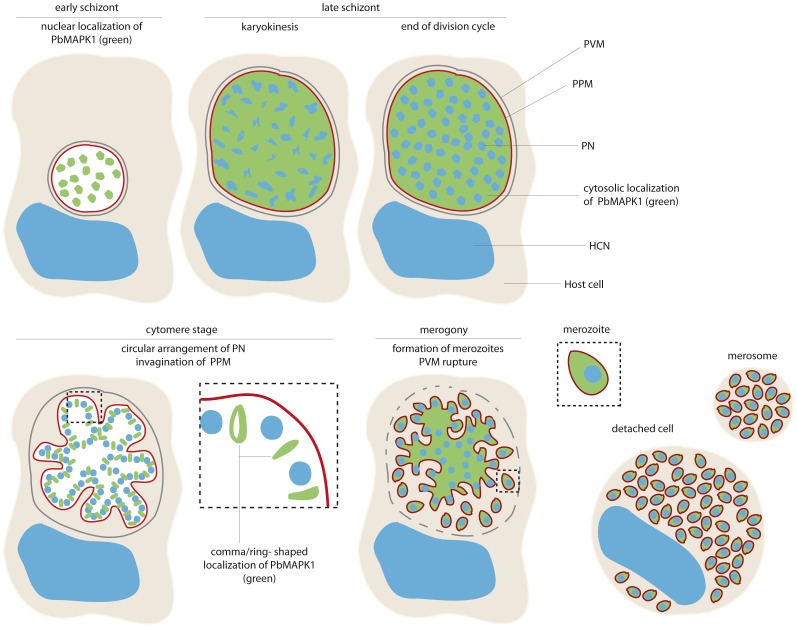
Schematic representation: stage-dependent subcellular localization of GFP-tagged MAPK1 in *P. berghei* liver stage parasites. While PbMAPK1 is observed inside the parasite nuclei at early schizont stage, at cytomere stage a distinct, comma/ring-shaped localization pattern occurs. PVM: parasitophorous vacuole membrane; PPM: parasite plasma membrane; PN: parasite nucleus; HCN: host cell nucleus.

In contrast to PbMAPK2, the PbMAPK1 protein localizes to fundamentally different subcellular sites in early and late liver stage parasites (summarized in **Figure 7**). During early schizogony, the kinase was detected inside the parasite nuclei with nuclear import being mediated by nuclear localization signals in the C-terminal extension domain of the kinase. When transiently expressed in a mammalian cell line, the PbMAPK1 C-terminal extension domain was found to accumulate in the nucleoli as was already reported previously for the *P. falciparum* MAPK1 full-length protein [18]. However, no subnuclear structures were seen in *P. berghei* parasites expressing GFP-tagged PbMAPK1, although the existence of a nucleolus was reported for *Plasmodium* parasites [24], [25]. While nuclear targeting is mediated by well-characterized motifs and the nuclear import/export machinery is at least partially conserved between mammalian cells and apicomplexan parasites [19], much less is known about nucleolar targeting. In mammalian cells, nucleolar accumulation of a protein is assumed to be induced mainly by specific interactions with constituents of the nucleolus, that is with rDNA, its transcripts or other nucleolar proteins [26].

In eukaryotic systems, activated MAPKs usually translocate to the nucleus where they regulate gene expression by phosphorylation of downstream targets like transcription factors and enzymes involved in chromatin remodeling [27]. Until now, no substrate proteins have been described for plasmodial MAPKs; potential candidates could be transcription factors like Myb [28], HMG [29] or the members of the ApiAP2 family recently shown to be involved in *Plasmodium* parasite development [30], [31].

Although the amino acid sequences of the C-terminal domains of PbMAPK1 and PfMAPK1 differ strikingly in length and composition, analysis of PfMAPK1 using the PSORTII program (http://psort.hgc.jp) reveals the occurrence of both putatively functional NLS motifs and a predicted coiled-coil domain in the C-terminal extension region of the *P. falciparum* enzyme. Similarly, basic stretches potentially mediating nuclear localization were detected in the *Toxoplasma gondii* MAPK1 homolog TgMAPK2 [32], [33] hinting towards a conserved function of the enzyme in different apicomplexan parasites.

At late schizont stage, GFP-tagged full-length PbMAPK1 no longer accumulated in the nuclei of liver stage parasites but was found evenly distributed throughout the parasite. Thus, nuclear targeting of the full-length protein is stage-specific and restricted to the early schizont stage when massive parasite growth and nuclear division is initiated. As soon as schizogony is complete, the parasite plasma membrane surrounding the syncytium starts to invaginate. After passing this phase referred to as cytomere stage, single infectious merozoites are formed (merogony) by organelle division and segregation and ongoing membrane invagination [34]. During the cytomere stage, GFP-tagged full-length PbMAPK1 was found in comma/ring-shaped structures in close proximity to the parasite’s nuclei and the invaginating parasite plasma membrane. In contrast, a GFP fusion protein of the PbMAPK1 C-terminal extension domain was found in the parasite nuclei throughout liver stage development and the isolated catalytic domain of the enzyme localized permanently to the parasite cytosol. Thus, both domains are necessary to promote re-localization of PbMAPK1 from the nucleus to the comma-shaped structures observed in cytomere stage parasites. A possible scenario could be (auto)phosphorylation of the PbMAPK1 enzyme triggering exposure of an nuclear export signal and/or silencing of nuclear localization signals. Indeed, subcellular localization of the mammalian MAPK ERK5 is influenced by a similar mechanism: here, activation of the enzyme by an upstream kinase induces a conformational change silencing a putative NES and exposing a bipartite NLS in the kinase’s C-terminal domain thereby promoting nuclear import of the protein ([35], [36]).

The localization of full-length PbMAPK1 to discrete extranuclear structures in proximity to the invaginating parasite plasma membrane in cytomere stage parasites may hint towards a function of the kinase in the cytokinesis events resulting in merozoite formation, for example in organelle fission and/or abscission. In mammalian cells, abscission takes place at a complex structure referred to as the midbody [37]. Here, antiparallel microtubule bundles interact with a plethora of structural and signaling proteins including several protein kinases [38] like MAPK kinase and the MAPKs ERK1/2 [39], [40], [41], [42] which also localize to the nucleus at earlier stages of cell division [40]. Furthermore, phosphoinositides like phosphatidyl-inositol-3-phosphate (PtdIns(3)P) and phosphatidyl-4,5-bisphosphate (PtdIns(4,5)P_2_) are enriched at the cleavage furrow/midbody of mammalian cells [43], [44], [45]. Thus, transgenic *P. berghei* parasites expressing GFP-tagged phosphoinositide binding domains like the pleckstrin-homology (PH) domain [46] and the Fab1/YOTB/Vac1/EEA1 (FYVE) domain [47] would be valuable tools for identification of putative midbody-like structures in liver stage parasites. Recently, expression of a GFP-2xFYVE fusion protein in apicomplexan parasites revealed localization of PtdIns(3)P in the food vacuole membrane and the apicoplast of *P. falciparum* blood stage parasites [48] and the apicoplast of *Toxoplasma gondii* [49].

The dynamic, stage-dependent localization pattern of PbMAPK1 was found to be independent of the kinase’s enzymatic activity and the integrity of the putative activation motif TDY. A similar observation was made previously for the atypical mammalian MAPK ERK7: here, both the wildtype and a kinase-dead mutant protein display a predominantly nuclear localization that is mediated by the protein’s C-terminal extension domain [50]. Several kinase activity-independent functions of a variety of different kinases have been described [51]. For example, ERK proteins can influence cytoskeletal organization, cell cycle regulation, and gene expression independently of their kinase activity by acting as scaffolding molecules, by allosterically modifiying other proteins, or even by direct binding to DNA [52].

While severe developmental defects have been observed for MAPK2 knockout parasites, blood stage parasites lacking MAPK1 showed normal blood and mosquito stage development [5], [12], [13], [14], [15]. Surprisingly, we also did not observe any apparent defects during *in vitro* liver stage development of PbMAPK1 knockout parasites: hepatocyte infection rates, phenotypic appearance of liver stage parasites and the capability of the parasites to successfully complete liver stage development by the formation of detached cells was unaltered when compared to wildtype parasites. Correspondingly, no change in the prepatency period after infection of mice with PbMAPK1 knockout sporozoites was reported by Tewari *et al.* [5]. In *P. falciparum* blood stage parasites, Dorin-Semblat *et al.* [12] observed elevated levels of the PfMAPK2 protein in PfMAPK1 knockout parasites. Thus, the loss of MAPK1 activity may be at least partially compensated for by the MAPK2 enzyme as was already observed for MAPK isoforms in the mammalian system [53]. Nevertheless, the different localization patterns of the plasmodial MAPK isoforms particularly during late liver stage development observed in our study suggest that other mechanisms for compensating for the lack of MAPK1 may exist in liver stage parasites. Alternatively, MAPK1 function may in fact not be essential for the parasite in the short-term but could influence parasite fitness in the long run.

## Materials and Methods

### Ethics Statement

All experiments involving mice were conducted in compliance with regulations created and approved by the ethical committee of Hamburg state authorities (no. FI 28/06), and all efforts were made to minimize suffering. Mosquito feeds were performed on mice anaesthetized with Ketavet/Domitor; mice were sacrificed using CO_2_.

### Sequences and Sequence Analysis

Coding sequences for the *P. berghei* and *P.falciparum* MAPK1 and MAPK2 proteins (Accession numbers: PBANKA_101330 (PbMAPK1), PBANKA_093370 (PbMAPK2), PF14_0294 (PfMAPK1), PF11_0147 (PfMAPK2)) were obtained from PlasmoDB (http://plasmodb.org/plasmo/). Prediction of nuclear localization signals and putative coiled- coil domains was performed using the PSORTII program accessible via http://psort.hgc.jp/.

### Transgenic Parasite Lines

For expression of a PbMAPK1-GFP fusion protein under control of the endogenous *pbmapk1* promoter, a 1257 bp fragment of the PbMAPK1 coding sequence was amplified using primers 5′-ATCCGCGGACAGAAGTCAATGAAAATAAAATACCAG-3′ and 5′-AATCCATGGAATATTTTTTCTTTTGTTTATAAAAATAATG-3′. The fragment was ligated into the vector pL0031 (obtained from MR4, deposited by A. Waters) using the SacII and NcoI restriction sites. Upon linearization with XbaI the plasmid was used for electroporation of *P. berghei* blood stage schizonts as described by Janse et al. [54] and selection was performed by pyrimethamine treatment of infected mice. Correct integration by single cross-over (resulting in an open reading frame encoding full length PbMAPK1 C-terminally fused to GFP) was confirmed by PCR analysis of genomic DNA (prepared with the NucleoSpin Blood QuickPure Kit, Macherey-Nagel) using the primer pair p1/p3 (5′ – ATTAACAGTTAGAAGAGGATTGCC –3′/5′-GCATCACCTTCACCCTCTCC-3′; 1999 bp). Detection of the wildtype *pbmapk1* locus was performed using the primer pair p1/p4 (5′ – ATTAACAGTTAGAAGAGGATTGCC–3′/5′-ATGCTAGCGATACATACATATTTATTTTCGAG-3′; 1931 bp); as a positive control, the PbMAPK1 ORF was amplified from genomic DNA using the primer pair p1/p2 (5′ – ATTAACAGTTAGAAGAGGATTGCC–3′/5′-ATGGATCCATATTTTTTCTTTTGTTTATAAAAATAATG-3′; 1888 bp).

Transgenic parasite lines expressing GFP fusion proteins of PbMAPK1 and PbMAPK2 wildtype and mutant constructs under the control of the constitutive EEF1alpha promoter or a liver stage specific promoter were generated in brief as follows (for a detailed description of all vector constructs see Table S1): Vector constructs for constitutive expression of fusion proteins under the control of the EEF1alpha promoter were generated based on the plasmid pL0017 (obtained from MR4, deposited by A. Waters). Constructs for liver stage-specific expression were produced using the plasmid pGFP_103464_ [17], a pL0017-based vector in which the constitutive EEF1alpha promoter has been replaced by a liver stage-specific promoter. Plasmids were linearized with ApaI and SacII and used for transfection of *P. berghei* blood stage schizonts as described by Janse *et al.* [54].

*Pb* ^con^GFP parasites constitutively expressing cytosolic GFP [55] were obtained from Chris Janse (Leiden University Medical Centre, The Netherlands).

### Construction of pEGFP-C2-PbMAPK1-CTD Expression Plasmids

An 828 bp fragment encoding the C-terminal domain of PbMAPK1 was amplified using the primer pair 5′ – AT*CTCGAG*TATTACAATACCAGTTGATGAAAGTAC –3′/5′ – AT*GGATCC*GATACATACATATTTATTTTCGAG –3′ (reverse primer binds in 3′UTR) and ligated into pEGFP-C2 (Clontech) using the XhoI and BamHI restriction sites to generate the plasmid pEGFP-C2-PbMAPK1-CTD. Based on this construct, deletion mutants were generated as summarized in Table S2.

### Transfection of HepG2 Cells

HepG2 cells (obtained from European Collection of Cell Cultures (ECACC)) were transfected by electroporation using the Nucleofector® system (Lonza) according to the manufacturer’s instructions. Briefly, 2×10^6^ cells were transfected with 3 µg plasmid DNA using Nucleofector Solution V and the T-028 program and seeded either in glass bottom dishes (35 mm diameter, 2.5×10^5^ cells per dish) for live cell imaging or on glass cover slips in 24 well plates (10^5^ cells/well) for immunofluorescence analysis.

### Infection of Mice and *Anopheles stephensi* Mosquitoes with *P. berghei*

Infection of 6–8 weeks old female NMRI mice (obtained either from Charles River Laboratories or bred in-house at the Bernhard Nocht Institute’s animal facility) with *P. berghei* was performed by i.p. injection of infected blood stabilates or fresh blood as described previously [56] or by i.v. injection of transfected blood stage schizonts. *Anopheles stephensi* mosquitoes were infected by blood meals on infected, anaesthetized mice.

### Infection of HepG2 Cells with *P. berghei*

For live cell imaging of transgenic *P. berghei* parasites, 10^5^ HepG2 cells were seeded in glass bottom dishes (35 mm diameter) the day prior to infection. Salivary glands of infected *A. stephensi* mosquitoes were collected on ice in standard HepG2 growth medium supplemented with amphotericin and disrupted using a pestle. Sporozoites were counted using a hemocytometer and applied to the HepG2 cells in standard HepG2 growth medium at a concentration of 2×10^4^–10^5^ sporozoites in 0.5 ml medium per dish. After a brief centrifugation (500 g, 5 min, RT), dishes were incubated for 2–3 h at 37°C, 5% CO_2_ before replacing the medium with 2 ml standard HepG2 growth medium.

### Live Cell Imaging

At indicated time points, live cell imaging was performed at a constant temperature of 37°C on an Olympus FV1000 confocal microscope using the Olympus Fluoview 1.7 b Software. Prior to imaging infected respectively transfected HepG2 cells were loaded with Hoechst 33342 (final concentration 1 µg/ml in standard HepG2 growth medium) to visualize host cell and parasite nuclei.

### Quantification of Comma/Ring-shaped Structures in Transgenic *P. berghei* Liver Stage Parasites

Both the number of comma/ring-shaped structures and the number of parasite nuclei were counted from confocal images of transgenic *P. berghei* parasites overexpressing GFP-tagged PbMAPK1 at 54 hpi and the ratio of number of comma/ring-shaped structures to number of nuclei was calculated. In a typical parasite at this stage, approximately 100–200 nuclei/confocal image are visible. Only images of parasites showing complete development of the characteristic comma/ring shaped localization pattern were used for quantification.

### RT-PCR Analysis of *P. berghei* Liver Stage Parasites

HepG2 cells were seeded in 24-well plates at a density of 5–7×10^4^ cells per well and infected with 1–2×10^4^ *P. berghei* sporozoites the following day. At indicated time points, infected cells were harvested and total RNA was isolated using the NucleoSpin RNAII Kit (Macherey-Nagel) according to the manufacturer’s instructions. cDNA was synthesized in a random-primed reverse transcriptase reaction and utilized as a template in PCR reactions using the following primer pairs: *pbmapk1* (358 bp): 5′ – TATTCACAGAGAGCATTGCCC –3′/5′ – TCCTTTTTCTTTGTTCCCTTG –3′; *pbmapk2* (220 bp): 5′ – ATTATTCCCGCACAGAAAACC –3′/5′ – AACCATCCAATCATCAAAAGG –3′. As a positive control, a 421 bp fragment of the *pbtubulin* cDNA was amplified using the primer pair 5′ – TGGAGCAGGAAATAACTGGG –3′/5′ – ACCTGACATAGCGGCTGAAA –3′. To rule out false positive results originating from gDNA contamination, samples containing no reverse transcriptase were processed in parallel.

### Determination of Sporozoite Infectivity and Parasite Conversion Rates

Successful completion of liver stage development is characterized by merozoite formation followed by PVM breakdown and merosome formation. *In vitro*, infected cells containing mature merozoites detach and can be found floating in the culture supernatant [1]. Thus, the ability of parasites to complete liver stage development can be quantified by counting the number of merozoite-containing detached cells in the culture supernatant. Therefore, HepG2 cells were seeded in 24-well plates on glass cover slips at a density of 5–7×10^4^ cells per well and infected with 1–2×10^4^ *P. berghei* sporozoites per well the following day. 24 hpi and 48 hpi, infected cells were fixed, permeabilized and stained with chicken anti-Exp1 and then either anti-chicken Cy2 or anti-chicken Alexa Fluor® 594 and DAPI as described below; numbers of parasites present at 24 hpi and 48 hpi were determined using fluorescence microscopy. At 65 hpi the supernatant of the remaining wells (containing detached cells) was transferred to fresh wells. After staining the nuclei with Hoechst 33342 (final concentration 1 µg/ml) detached cells were quantified using fluorescence microscopy (Axiovert 200, Zeiss). Infectivity was calculated as the percentage of sporozoites having successfully established host cell infection at 24 hpi; parasite conversion rate was calculated as the ratio of the number of detached cells to the number of parasites present at 24 hpi. For graphical representation, conversion rates were normalized with respect to the conversion rate obtained for WT parasites.

### Immunofluorescence Analysis

Infected HepG2 cells were fixed and permeabilized at the indicated time points using 4% paraformaldehyde (20 min, RT) and icecold methanol (5 min, −20°C); for fibrillarin-costaining, transfected cells were fixed and permeabilized by a 10 min incubation in icecold methanol followed by a 10 min incubation in icecold aceton. After blocking for 1 h at room temperature with 10% FCS/PBS, primary antibodies were applied in 10% FCS/PBS for 2 h at room temperature followed by secondary antibody incubation in 10% FCS/PBS for 1 h at room temperature. Cover slips were mounted onto glass slides with DAKO Fluorescent Mounting Medium. Primary antibodies used were: chicken anti-Exp1 (1∶1000), mouse anti-GFP (1∶1000, Molecular Probes) and rabbit anti-fibrillarin (1∶1000, Abcam). Secondary antibodies used were: goat anti-chicken Cy2 (1∶400, Dianova), goat anti-chicken IgG Alexa Fluor® 594 (1∶5000, Molecular Probes), goat anti-mouse IgG Alexa Fluor® 488 (1∶5000, Molecular Probes) and donkey anti-rabbit Alexa Fluor® 594 (1∶5000, Invitrogen). Parasite and host cell nuclei were visualized using DAPI at a final concentration of 1 µg/ml added to the secondary antibody solution.

### Western Blot Analysis of Mixed Blood Stage Parasite Protein Extracts

Blood from *P. berghei*-infected mice was collected by heart puncture. Blood cells were washed three times with icecold PBS and then lysed by incubation in 0.05% saponin/PBS for 9 min on ice. After centrifugation (1 min, 11000 g), the parasite pellet was washed twice with icecold PBS before resuspension in SDS-PAGE Loading Buffer. Protein samples were separated on 12% SDS-PAGE gels and blotted onto nitrocellulose. Protein detection was performed using mouse anti-GFP (1∶2000, Roche Diagnostics)/goat anti-mouse HRP (1∶1000, Pierce) antibodies and SuperSignal West femto (Pierce).

### Statistical Data Analysis

Quantitative data are presented as mean +/− SD. Student’s t test was applied to obtain p values indicating statistical significance.

## Supporting Information

Figure S1 ***Gfp*** **-tagging of the endogenous** ***pbmapk1*** **gene by homologous recombination (single cross-over). (A) Schematic representation of the plasmid construct used for GFP-tagging and the resulting recombined** ***pbmapk1*** **-locus.** A 1257 bp fragment of the PbMAPK1 ORF was amplified by PCR and ligated into pL0031. The plasmid was linearized with XbaI and used for transfection of *P. berghei* blood stage schizonts. Recombinant parasites were selected by pyrimethamine treatment of infected mice. Dhfr: *T. gondii* Dihydrofolate-Reductase. **(B) Confirmation of correct integration by PCR analysis.** Genomic DNA of *P. berghei* WT and *Pb* ^end^PbMAPK1-GFP parasites was prepared from blood stage parasites and PCR analysis was performed using the primer pairs indicated in (A). **(C) Western blot analysis of** ***Pb*** **^end^PbMAPK1-GFP blood stage parasites.** A saponin extract was prepared from infected mouse blood, separated by SDS-PAGE and blotted onto nitrocellulose. Detection was performed using mouse anti-GFP/anti-mouse HRP. Molecular weight of marker proteins: kDa; expected molecular weight of PbMAPK1-GFP: 97 kDa. **(D) Live cell imaging of** ***Pb*** **^end^PbMAPK1-GFP blood stage parasites.** Infected erythrocytes were stained using Hoechst 33342 and microscopic analysis was performed by epifluorescence microscopy. Scale bar: 5 µm.
(TIF)

Figure S2 **The stage-specific localization of PbMAPK1 is independent of the integrity of the DFG-motif, the TDY activation motif and the putative coiled-coil domain.** HepG2 cells were infected with *Pb* ^con^PbMAPK1(D178A)-GFP, *Pb* ^con^PbMAPK1(T198A/Y200A)-GFP, *Pb* ^con^GFP-PbMAPK1(T198A/Y200A), or *Pb* ^LS^PbMAPK1Δcc-GFP parasites. Confocal live cell imaging was performed at 30 hpi (early schizont stage; *Pb* ^con^PbMAPK1(D178A)-GFP, *Pb* ^con^GFP-PbMAPK1(T198A/Y200A), *Pb* ^LS^PbMAPK1Δcc-GFP), and 54 hpi (cytomere stage; *Pb* ^con^PbMAPK1(D178A)-GFP, *Pb* ^con^PbMAPK1(T198A/Y200A)-GFP, *Pb* ^LS^PbMAPK1Δcc-GFP). Scale bar: 5 µm; catD (catalytic domain); CTD (C-terminal domain); cc (coiled-coil); aa (amino acids).
(TIF)

Figure S3 **Live imaging of double-fluorescent mCherry-PbMAPK1-GFP parasites.** HepG2 cells were infected with parasites constitutively expressing PbMAPK1 with an N-terminal mCherry- and a C-terminal GFP-tag (*Pb* ^con^mCherry-PbMAPK1-GFP). Live cell imaging was performed at different developmental stages (early schizont, cytomere, merozoites). Infected cells were loaded with Hoechst 33342 to visualize host cell and parasite nuclei. Scale bars: 10 µm.
(TIF)

Figure S4 **Characterization of PbMAPK1 NLSs in HepG2 cells.** HepG2 cells were transiently transfected with pEGFP-C2 based plasmids encoding GFP-PbMAPK1-CTD deletion constructs (for schematic overview see Figure 5). 24 hours post transfection, nuclei were stained using Hoechst 33342 and live cell imaging was performed. Control: mock transfected HepG2 cells (pEGFP-C2). Scale bar: 10 µm.
(TIF)

Figure S5 **The C-terminal domain of PbMAPK1 localizes to the nucleolus of HepG2 cells.** HepG2 cells were transfected with pEGFP-C2-PbMAPK1-CTD. 24 hours post transfection cells were fixed, permeabilized and stained with rabbit anti-fibrillarin/anti-rabbit Alexa Fluor® 594, mouse anti-GFP/anti-mouse Cy2 and DAPI. In merged pictures, DAPI-stained nuclei are depicted in blue, GFP-PbMAPK1-CTD is shown in green and fibrillarin in red. Scale bar: 10 µm.
(TIF)

Figure S6 ***P. berghei*** **MAPK1 knockout parasites show phenotypically normal liver stage development. (A)** HepG2 cells were infected with defined numbers of *P. berghei* WT and MAPK1 knockout sporozoites, respectively. 24 hpi, cells were fixed and stained with chicken anti-Exp1/anti-chicken Cy2 and DAPI. Using fluorescence microscopy, parasites were counted and infectivity was calculated as the percentage of sporozoites having successfully established host cell infection (n = 3 for both WT and MAPK1 knockout; results normalized on WT control; n.s. = not significant according to Student’s T test). **(B)** The parasites’ ability to successfully complete *in* *vitro* liver stage development was assayed by counting detached cells in the supernatant at 65 hpi (n = 3, results normalized on WT control). **(C)** HepG2 cells were infected with *P. berghei* MAPK1 knockout sporozoites. 54 hpi, infected cells were fixed and stained with chicken anti-Exp1/anti-chicken Cy2 and DAPI; scale bar: 10 µm.
(TIF)

Figure S7 **Subcellular localization of GFP-PbMAPK2.** HepG2 cells were infected with *Pb* ^con^GFP-PbMAPK2 parasites. Live cell imaging was performed at different developmental stages (early schizont, cytomere, merozoites). Infected cells were stained with Hoechst 33342 to visualize host cell and parasite nuclei. Scale bars: 10 µm.
(TIF)

Table S1 **Vectors for generation of transgenic parasites expressing PbMAPK1 and PbMAPK2 fusion proteins.**
(PDF)

Table S2 **Vectors for expression of GFP-tagged PbMAPK1-CTD deletion constructs in HepG2 cells.**
(PDF)

Movie S1 **Time course of subcellular localization of PbMAPK1-GFP.** HepG2 cells were infected with *Pb* ^con^PbMAPK1(D178A)-GFP parasites. Parasite development from late schizont stage to merozoite formation was monitored starting at 48 hpi.
(AVI)

Movie S2 **Spinning disc microscopy (z stack).** HepG2 cells were infected with *Pb* ^con^PbMAPK1(D178A)-GFP parasites. 54 hpi localization of the GFP fusion protein was monitored by spinning disk microscopy.
(MOV)
