# Supplementary figures and images for: Plasmodium berghei MAPK1 Displays Differential and Dynamic Subcellular Localizations during Liver Stage Development
Source: PLoS One. 2013 Mar 27;8(3):e59755. doi: 10.1371/journal.pone.0059755 (PMC3609774; doi:10.1371/journal.pone.0059755)

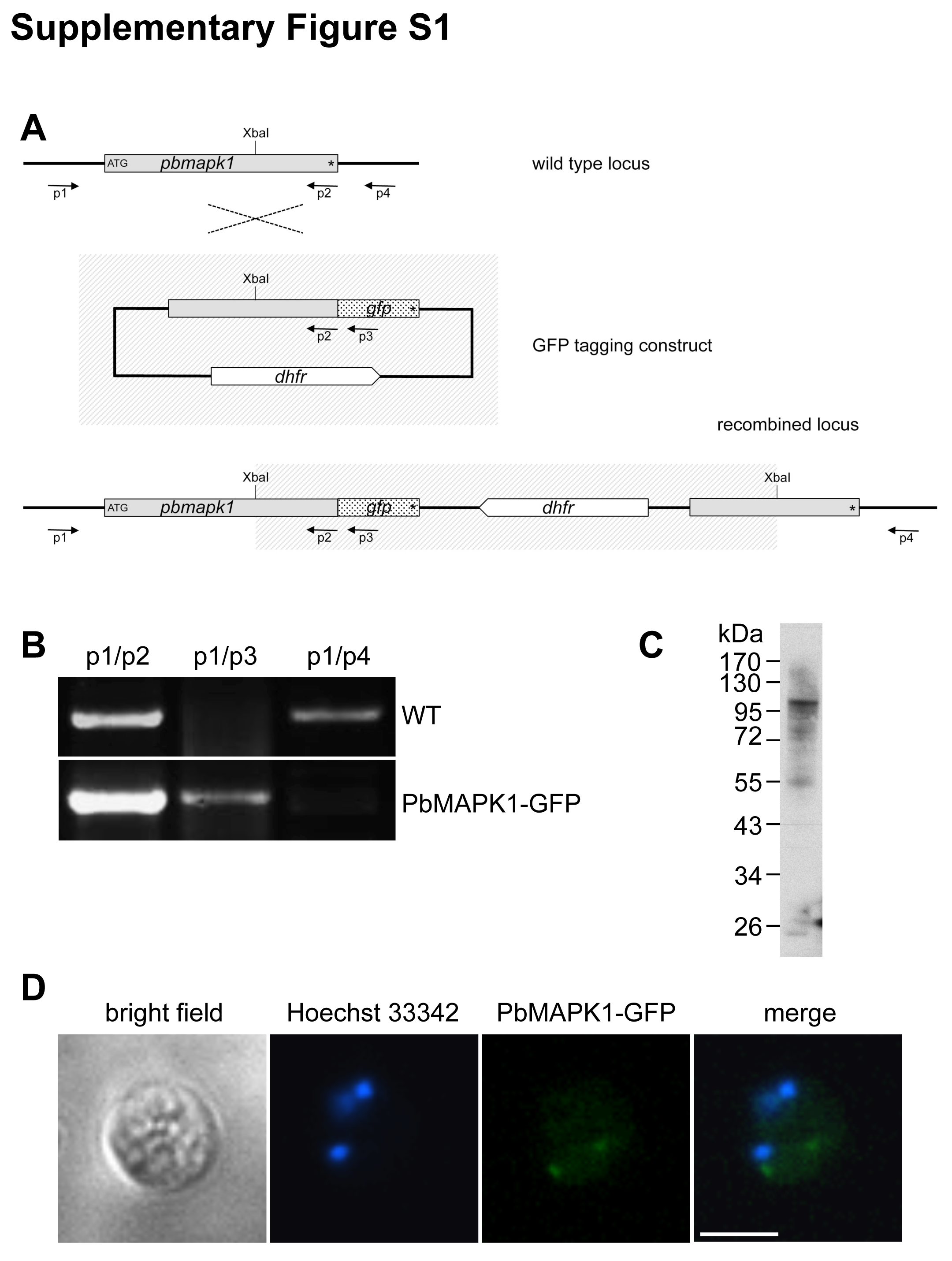

Supplement: Figure S1 — Gfp -tagging of the endogenous pbmapk1 gene by homologous recombination (single cross-over). (A) Schematic representation of the plasmid construct used for GFP-tagging and the resulting recombined pbmapk1 -locus. A 1257 bp fragment of the PbMAPK1 ORF was amplified by PCR and ligated into pL0031. The plasmid was linearized with XbaI and used for transfection of P. berghei blood stage schizonts. Recombinant parasites were selected by pyrimethamine treatment of infected mice. Dhfr: T. gondii Dihydrofolate-Reductase. (B) Confirmation of correct integration by PCR analysis. Genomic DNA of P. berghei WT and Pb endPbMAPK1-GFP parasites was prepared from blood stage parasites and PCR analysis was performed using the primer pairs indicated in (A). (C) Western blot analysis of Pb endPbMAPK1-GFP blood stage parasites. A saponin extract was prepared from infected mouse blood, separated by SDS-PAGE and blotted onto nitrocellulose. Detection was performed using mouse anti-GFP/anti-mouse HRP. Molecular weight of marker proteins: kDa; expected molecular weight of PbMAPK1-GFP: 97 kDa. (D) Live cell imaging of Pb endPbMAPK1-GFP blood stage parasites. Infected erythrocytes were stained using Hoechst 33342 and microscopic analysis was performed by epifluorescence microscopy. Scale bar: 5 µm. (TIF) [file pone.0059755.s001.tif]

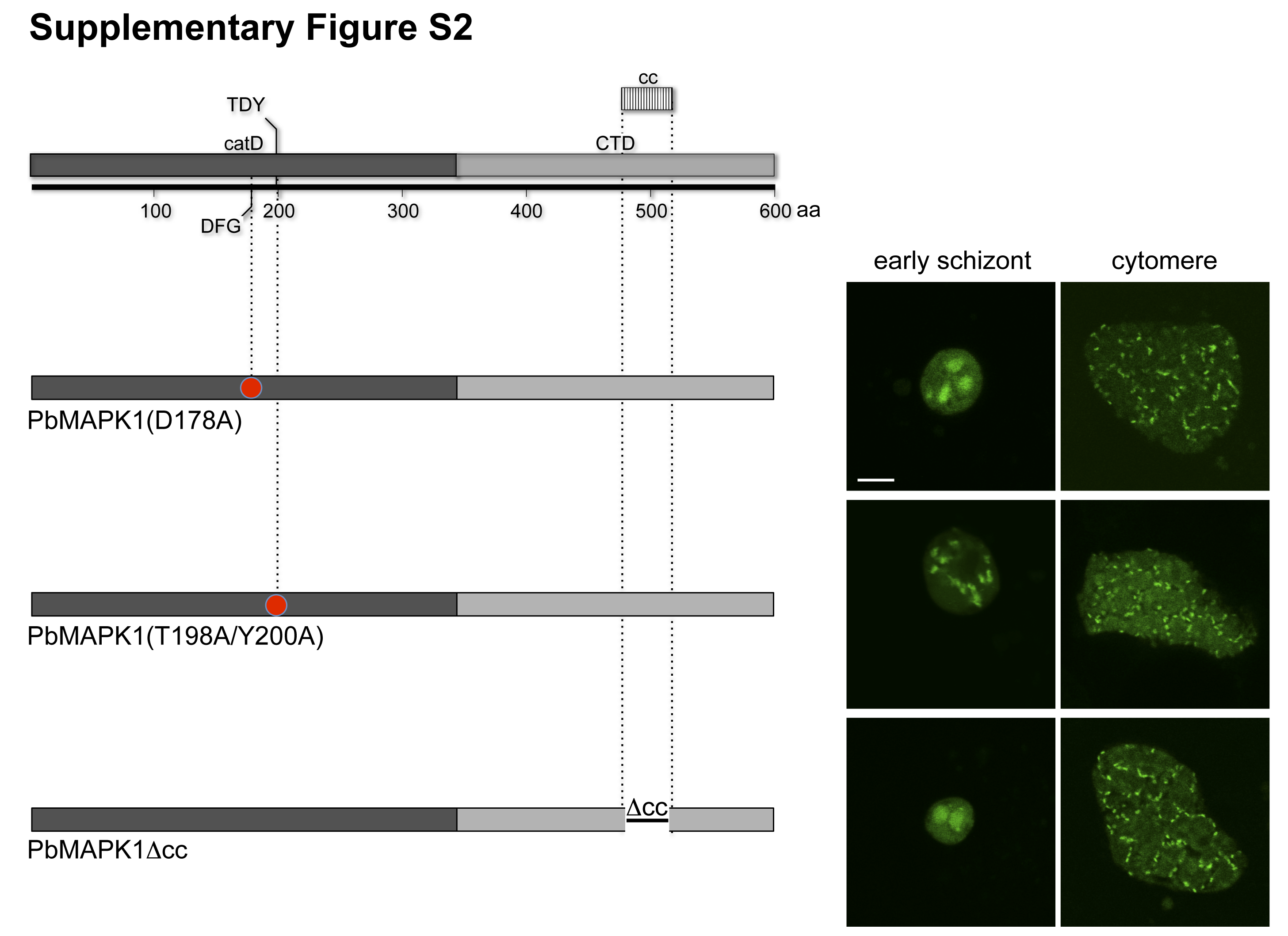

Supplement: Figure S2 — The stage-specific localization of PbMAPK1 is independent of the integrity of the DFG-motif, the TDY activation motif and the putative coiled-coil domain. HepG2 cells were infected with Pb conPbMAPK1(D178A)-GFP, Pb conPbMAPK1(T198A/Y200A)-GFP, Pb conGFP-PbMAPK1(T198A/Y200A), or Pb LSPbMAPK1Δcc-GFP parasites. Confocal live cell imaging was performed at 30 hpi (early schizont stage; Pb conPbMAPK1(D178A)-GFP, Pb conGFP-PbMAPK1(T198A/Y200A), Pb LSPbMAPK1Δcc-GFP), and 54 hpi (cytomere stage; Pb conPbMAPK1(D178A)-GFP, Pb conPbMAPK1(T198A/Y200A)-GFP, Pb LSPbMAPK1Δcc-GFP). Scale bar: 5 µm; catD (catalytic domain); CTD (C-terminal domain); cc (coiled-coil); aa (amino acids). (TIF) [file pone.0059755.s002.tif]

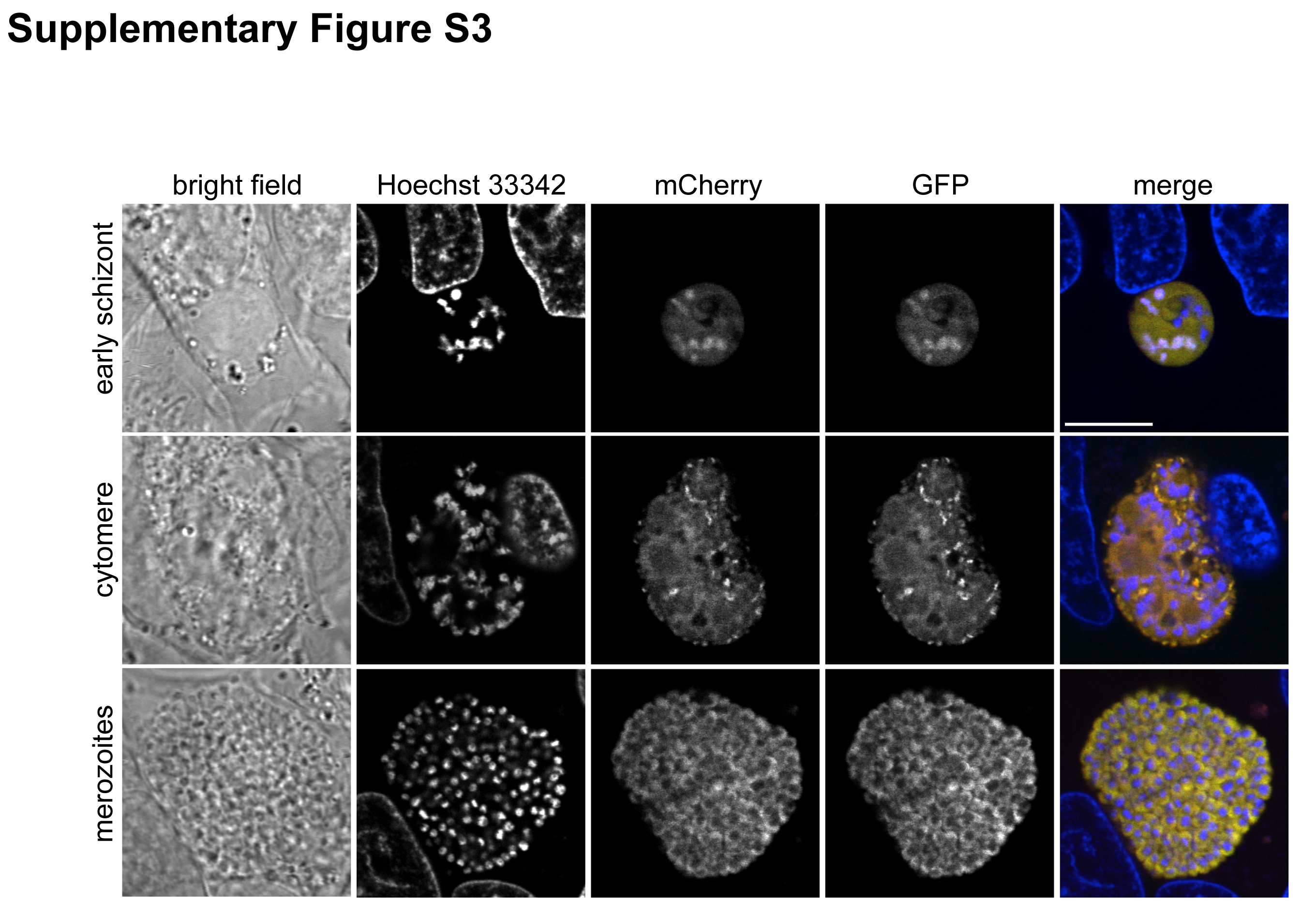

Supplement: Figure S3 — Live imaging of double-fluorescent mCherry-PbMAPK1-GFP parasites. HepG2 cells were infected with parasites constitutively expressing PbMAPK1 with an N-terminal mCherry- and a C-terminal GFP-tag (Pb conmCherry-PbMAPK1-GFP). Live cell imaging was performed at different developmental stages (early schizont, cytomere, merozoites). Infected cells were loaded with Hoechst 33342 to visualize host cell and parasite nuclei. Scale bars: 10 µm. (TIF) [file pone.0059755.s003.tif]

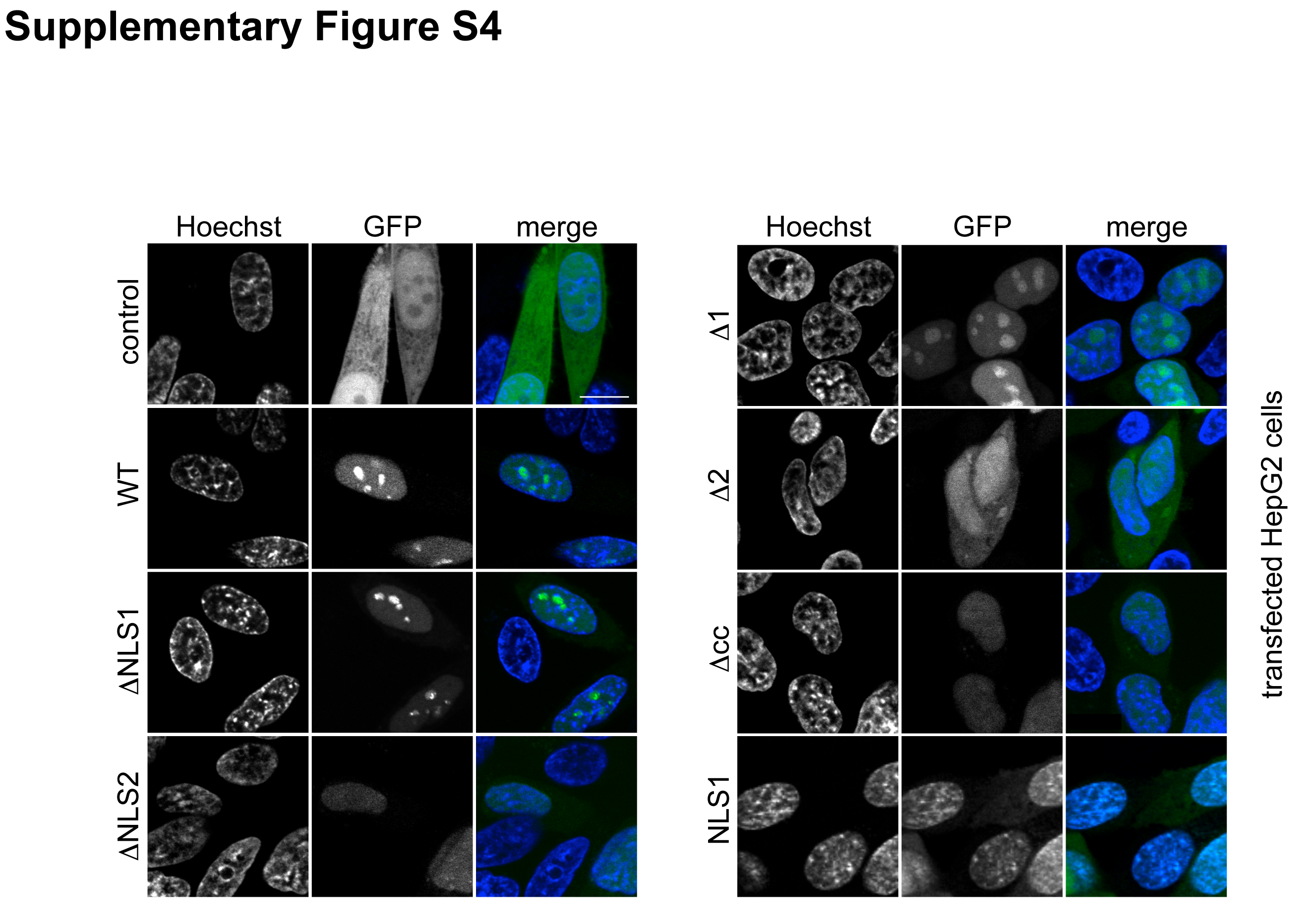

Supplement: Figure S4 — Characterization of PbMAPK1 NLSs in HepG2 cells. HepG2 cells were transiently transfected with pEGFP-C2 based plasmids encoding GFP-PbMAPK1-CTD deletion constructs (for schematic overview see Figure 5). 24 hours post transfection, nuclei were stained using Hoechst 33342 and live cell imaging was performed. Control: mock transfected HepG2 cells (pEGFP-C2). Scale bar: 10 µm. (TIF) [file pone.0059755.s004.tif]

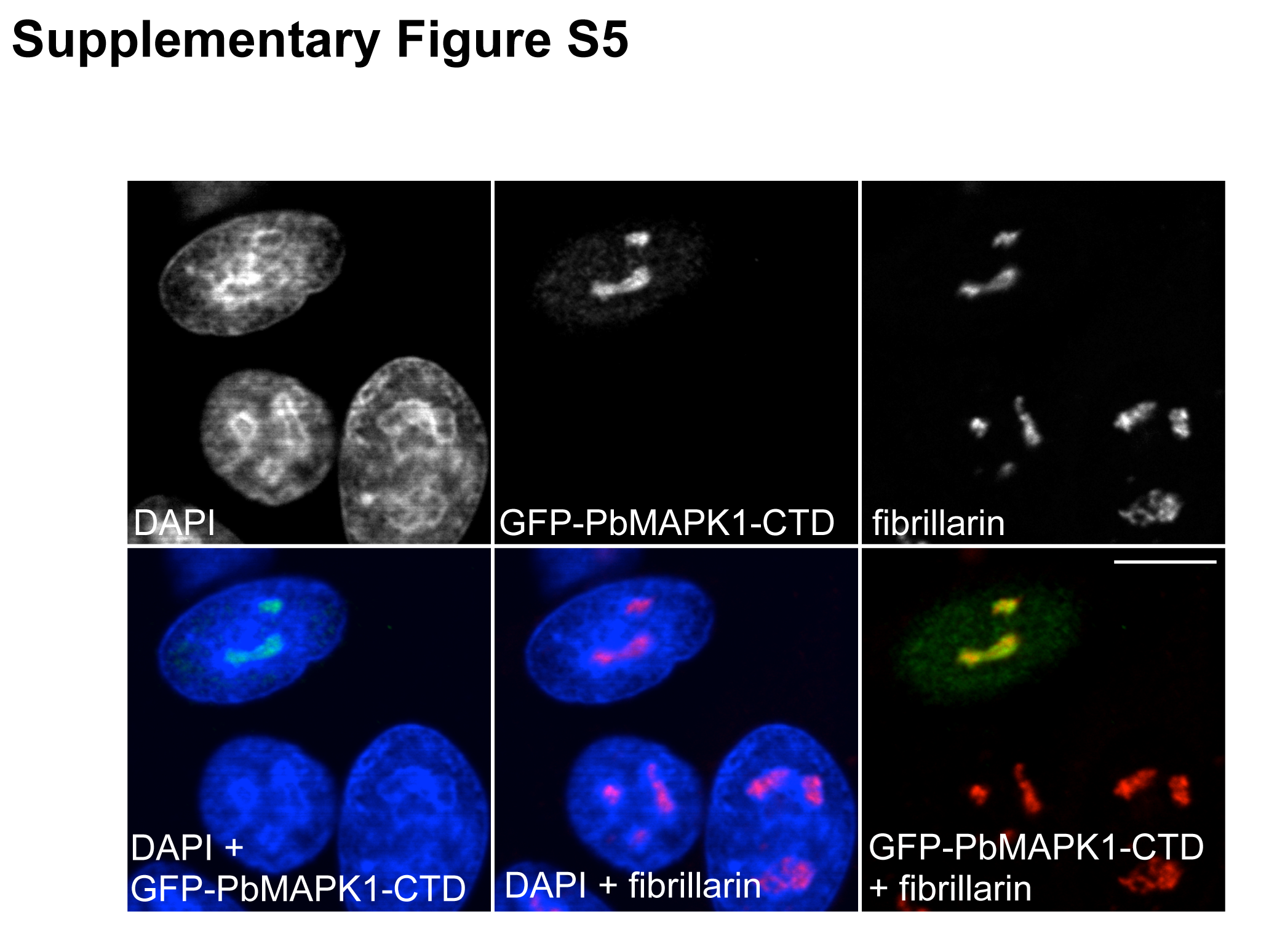

Supplement: Figure S5 — The C-terminal domain of PbMAPK1 localizes to the nucleolus of HepG2 cells. HepG2 cells were transfected with pEGFP-C2-PbMAPK1-CTD. 24 hours post transfection cells were fixed, permeabilized and stained with rabbit anti-fibrillarin/anti-rabbit Alexa Fluor® 594, mouse anti-GFP/anti-mouse Cy2 and DAPI. In merged pictures, DAPI-stained nuclei are depicted in blue, GFP-PbMAPK1-CTD is shown in green and fibrillarin in red. Scale bar: 10 µm. (TIF) [file pone.0059755.s005.tif]

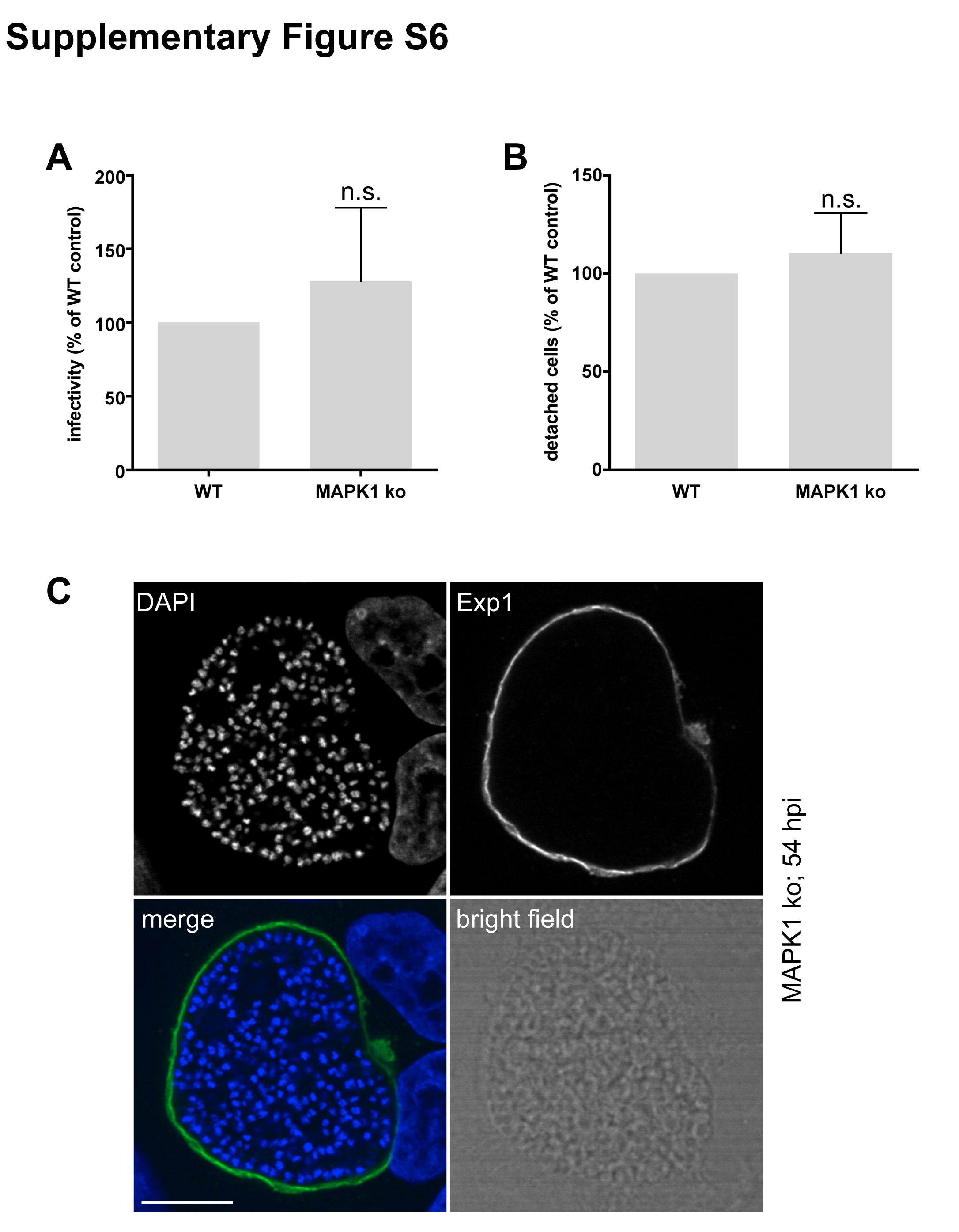

Supplement: Figure S6 — P. berghei MAPK1 knockout parasites show phenotypically normal liver stage development. (A) HepG2 cells were infected with defined numbers of P. berghei WT and MAPK1 knockout sporozoites, respectively. 24 hpi, cells were fixed and stained with chicken anti-Exp1/anti-chicken Cy2 and DAPI. Using fluorescence microscopy, parasites were counted and infectivity was calculated as the percentage of sporozoites having successfully established host cell infection (n = 3 for both WT and MAPK1 knockout; results normalized on WT control; n.s. = not significant according to Student’s T test). (B) The parasites’ ability to successfully complete in vitro liver stage development was assayed by counting detached cells in the supernatant at 65 hpi (n = 3, results normalized on WT control). (C) HepG2 cells were infected with P. berghei MAPK1 knockout sporozoites. 54 hpi, infected cells were fixed and stained with chicken anti-Exp1/anti-chicken Cy2 and DAPI; scale bar: 10 µm. (TIF) [file pone.0059755.s006.tif]

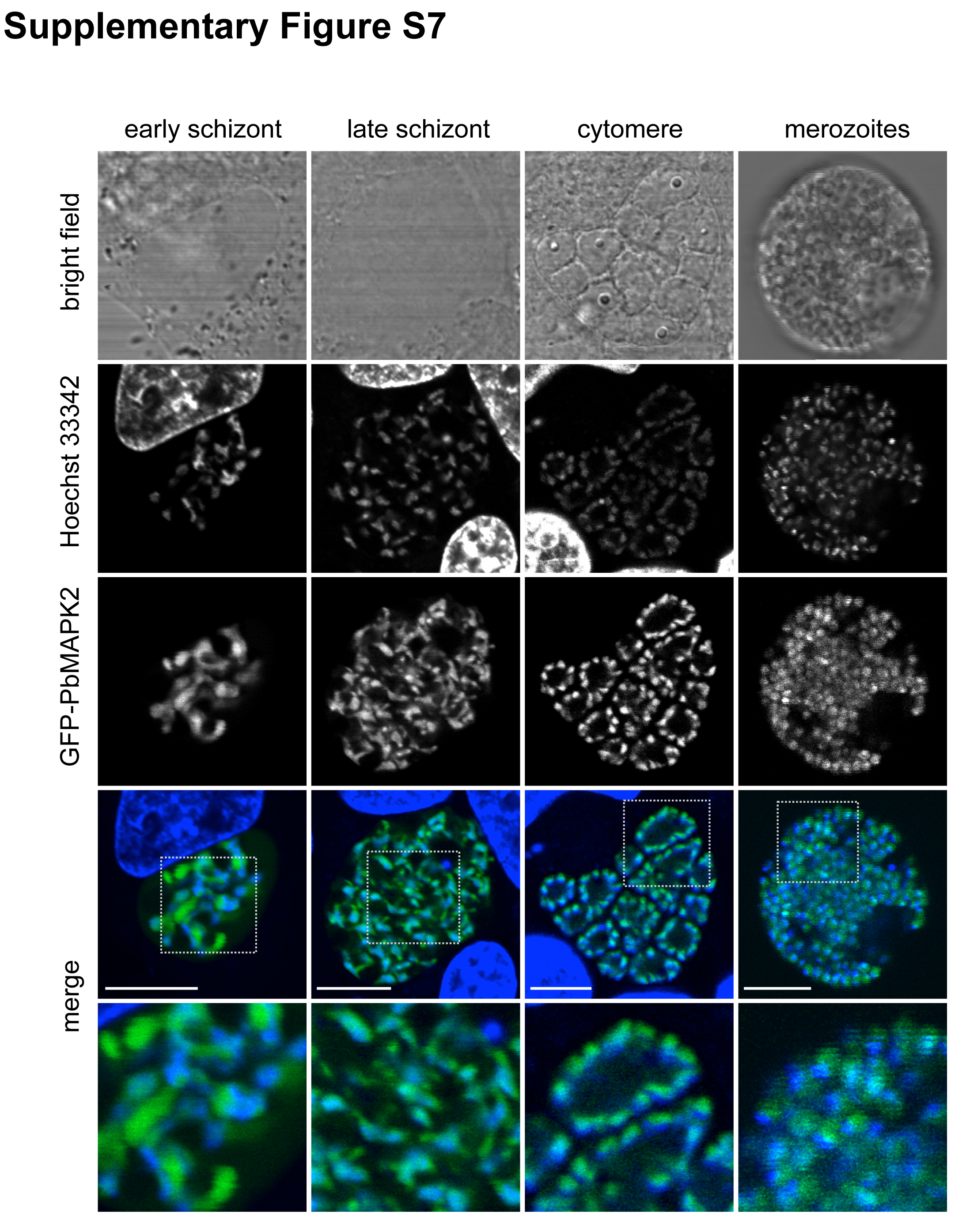

Supplement: Figure S7 — Subcellular localization of GFP-PbMAPK2. HepG2 cells were infected with Pb conGFP-PbMAPK2 parasites. Live cell imaging was performed at different developmental stages (early schizont, cytomere, merozoites). Infected cells were stained with Hoechst 33342 to visualize host cell and parasite nuclei. Scale bars: 10 µm. (TIF) [file pone.0059755.s007.tif]
