# Supplementary material for: Plasmodium berghei MAPK1 Displays Differential and Dynamic Subcellular Localizations during Liver Stage Development
Source: PLoS One. 2013 Mar 27;8(3):e59755. doi: 10.1371/journal.pone.0059755 (PMC3609774; doi:10.1371/journal.pone.0059755)
Supplement: Table S1 — Vectors for generation of transgenic parasites expressing PbMAPK1 and PbMAPK2 fusion proteins. (PDF) [file pone.0059755.s008.pdf]

Supplementary Table ST1: Vectors for generation of transgenic parasites expressing PbMAPK1 and PbMAPK2 fusion proteins

transgenic parasite line/fusion protein	vector	promoter	MW (kDa)	oligonucleotides used (5’ → 3’)	cloned in / via restriction sites
Pb^{con}PbMAPK1-GFP	pL0017-MCS1-PbMAPK1	EF1α	97	amplification of fragment:<br>AT*GCGGCCGC*ATGGAGAGAGAAAAACAGAAGAAAACTC/<br>AT*GGATCC*ATATTTTTTCTTTTGTTTATAAAAATAATG	pL0017-MCS1; NotI/BamHI
Pb^{LS}PbMAPK1-GFP	pL0017.1.1-PbMAPK1	liver stage-specific		amplification of fragment:<br>AT*GGATCC*ATGGAGAGAGAAAAACAGAAGAAAACTC/<br>AT*GGATCC*ATATTTTTTCTTTTGTTTATAAAAATAATG	pL0017.1.1; BamHI
Pb^{LS}PbMAPK1-V5	pL0017.1.7-PbMAPK1		72	amplification of fragment:<br>AT*GCGGCCGC*ATGGAGAGAGAAAAACAGAAGAAAACTC/<br>AT*GCTAGC*GCATATTTTTTCTTTTGTTTATAAAAATAATG	pL0017.1.7; NotI/AvrII (compatible to NheI)
Pb^{con}GFP-PbMAPK1	pL0017-GFP-N-PbMAPK1	EF1α	97	amplification of fragment:<br>AT*GCTAGC*ATGGAGAGAGAAAAACAGAAGAAAACTC/<br>AT*GCTAGC*GATACATACATATTTATTTTCGAG (primes in 3’UTR)	pL0017-GFP-N; XbaI (compatible ends to NheI)
Pb^{con}PbMAPK1(T198A/Y200A)-GFP	pL0017-MCS1-PbMAPK1(T198A/Y200A)	EF1α	97	amplification of fragment:<br>AT*GCGGCCGC*ATGGAGAGAGAAAAACAGAAGAAAACTC/<br>AT*GGATCC*ATATTTTTTCTTTTGTTTATAAAAATAATG<br>site-directed mutagenesis:<br>AATACCAGTATTAGCAGATGCTGTAGCA*ACGCGT*TGGTATAAGAGC/<br>GCTCTATACCA*ACGCGT*TGCTACAGCATCTGCTAATACTGGTATT	pL0017-MCS1; NotI/BamHI

Pb^{con}GFP-PbMAPK1(T198A/Y200A)	pL0017-GFP-N-PbMAPK1(T198A/Y200A)	EF1α	97	amplification of fragment:<br>AT*GCTAGC*ATGGAGAGAGAAAAACAGAAGAAAACTC/<br>AT*GCTAGC*GATACATACATATTTATTTTCGAG (primes in 3'UTR)<br>site-directed mutagenesis:<br>AATACCAGTATTAGCAGATGCTGTAGCA*ACGCGT*TGGTATAAGAGC/<br>GCTCTATACCA*ACGCGT*TGCTACAGCATCTGCTAATACTGGTATT	pL0017-GFP-N; XbaI (compatible ends to NheI)
Pb^{con}PbMAPK1(D178A)-GFP	pL0017-MCS1-PbMAPK1(D178A)	EF1α	97	amplification of fragment:<br>AT*GCGGCCGC*ATGGAGAGAGAAAAACAGAAGAAAACTC/<br>AT*GGATCC*ATATTTTTTCTTTTGTTTATAAAAATAATG<br>site-directed mutagenesis:<br>GAATGCCATTTAAAAATAT*TGCGCA*TTTGGATTGGCGCGAAG/<br>CTTCGCGCCAATCCAAA*TGCGCA*TATTTTTAAATGGCATTC	pL0017-MCS1; NotI/BamHI
Pb^{con}GFP-PbMAPK1(D178A)	pL0017-GFP-N-PbMAPK1(D178A)	EF1α	97	amplification of fragment:<br>AT*GCTAGC*ATGGAGAGAGAAAAACAGAAGAAAACTC/<br>AT*GCTAGC*GATACATACATATTTATTTTCGAG (primes in 3'UTR)<br>site-directed mutagenesis:<br>GAATGCCATTTAAAAATAT*TGCGCA*TTTGGATTGGCGCGAAG/<br>CTTCGCGCCAATCCAAA*TGCGCA*TATTTTTAAATGGCATTC	pL0017-GFP-N; XbaI (compatible ends to NheI)
Pb^{con}PbMAPK1-Δcc -GFP	pL0017-MCS1-PbMAPK1-Δcc	EF1α	93	amplification of fragment:<br>AT*GCGGCCGC*ATGGAGAGAGAAAAACAGAAGAAAACTC/<br>AT*GGATCC*ATATTTTTTCTTTTGTTTATAAAAATAATG<br>deletion (fusion PCR):<br>GCTCACAATATTTTAAAACAAATACAGTAAATTGCTATG/<br>CTGTATTTGTTTTAAAATATTGTGAGCTGTTTTCTCTGC	pL0017-MCS1; NotI/BamHI
Pb^{LS}PbMAPK1-Δcc -GFP	pL0017.1.1-PbMAPK1-Δcc	liver stage-specific		amplification of fragment:<br>AT*GGATCC*ATGGAGAGAGAAAAACAGAAGAAAACTC/<br>AT*GGATCC*ATATTTTTTCTTTTGTTTATAAAAATAATG<br>deletion (fusion PCR):<br>GCTCACAATATTTTAAAACAAATACAGTAAATTGCTATG/<br>CTGTATTTGTTTTAAAATATTGTGAGCTGTTTTCTCTGC	pL0017.1.1; BamHI
Pb^{LS}PbMAPK1-Δcc -V5	pL0017.1.7-PbMAPK1-Δcc		68	amplification of fragment:<br>AT*GCGGCCGC*ATGGAGAGAGAAAAACAGAAGAAAACTC/<br>AT*GCTAGC*GCATATTTTTTCTTTTGTTTATAAAAATAATG<br>deletion (fusion PCR):<br>GCTCACAATATTTTAAAACAAATACAGTAAATTGCTATG/<br>CTGTATTTGTTTTAAAATATTGTGAGCTGTTTTCTCTGC	pL0017.1.7; NotI/AvrII (compatible to NheI)

Pb^{con}GFP-PbMAPK1-Δcc	pL0017-GFP-N-PbMAPK1-Δcc	EF1α	93	amplification of fragment:<br>AT*GCTAGC*ATGGAGAGAGAAAAACAGAAGAAAACTC/<br>AT*GCTAGC*GATACATACATATTTATTTTCGAG (primes in 3'UTR)<br>deletion (fusion PCR):<br>GCTCACAATATTTTAAAACAAATACAGTAAATTGCTATG/<br>CTGTATTTGTTTTAAAATATTGTGAGCTGTTTTCTCTGC	pL0017-GFP-N; XbaI (compatible ends to NheI)
Pb^{con}mCherry-PbMAPK1-GFP	pL0017-MCS1-mCh-PbMAPK1	EF1α	126	amplification of mCherry coding sequence:<br>GA*GCGGCCGC*ATGGTGAGCAAGGGCGAGGAG/AA*GCGGCCGC*CC<br>CCCTTGTACAGCTCGTCCATGCC	pL0017-MCS1-PbMAPK1; NotI
Pb^{con}PbMAPK1-catD(T198A/Y200A)-GFP	pL0017-PbMAPK1-catD(T198A/Y200A)	EF1α	68	amplification of fragment:<br>AT*GGATCC*ATGGAGAGAGAAAAACAGAAGAAAACTC/<br>AT*GGATCC*AATATGTTTGCAAATTGGTTCCTC<br>site-directed mutagenesis:<br>AATACCAGTATTAGCAGATGCTGTAGCA*ACGCGT*TGGTATAAGAGC/<br>GCTCTATACCA*ACGCGT*TGCTACAGCATCTGCTAATACTGGTATT	pL0017; BamHI
Pb^{con}PbMAPK1- catD(D178A)-GFP	pL0017-PbMAPK1-katD(D178A)	EF1α	68	amplification of fragment:<br>AT*GGATCC*ATGGAGAGAGAAAAACAGAAGAAAACTC/<br>AT*GGATCC*AATATGTTTGCAAATTGGTTCCTC<br>site-directed mutagenesis:<br>GAATGCCATTTAAAAATA*TGCGCA*TTTGGATTGGCGCGAAG/<br>CTTCGCGCCAATCCAAA*TGCGCA*TATTTTTAAATGGCATTC	pL0017; BamHI
Pb^{con}GFP-PbMAPK1-CTD	pL0017-GFP-N-PbMAPK1-CTD	EF1α	59	amplification of fragment:<br>AT*GCTAGC*ATTACAATACCAGTTGATGAAAGTAC/<br>AT*GCTAGC*GATACATACATATTTATTTTCGAG (primes in 3'UTR)	pL0017-GFP-N; XbaI (compatible ends to NheI)
Pb^{con}GFP-PbMAPK1-CTD-Δ1	pL0017-GFP-N-PbMAPK1-CTD-Δ1	EF1α	54	amplification of fragment (XbaI site in PbMAPK1 ORF was used for cloning):<br>ATTCACAGAGAGCATTGCCCAG/<br>AT*TCTAGA*GATACATACATATTTATTTTCGAG (primes in 3'UTR)	pL0017-GFP-N; XbaI

Pb^{con}GFP-PbMAPK1-CTD-Δ2	pL0017-GFP-N-PbMAPK1-CTD-Δ2	EF1α	45	amplification of fragment:<br>AT*TCTAGA*TCTAATTCCCCAAATACACCAAATGC/<br>AT*TCTAGA*GATACATACATATTTATTTTCGAG (primes in 3'UTR)	pL0017-GFP-N; XbaI
Pb^{con}GFP-PbMAPK1-CTD-Δcc	pL0017-GFP-N-PbMAPK1-CTD-Δcc	EF1α	53	amplification of fragment:<br>AT*GCTAGC*ATTACAATACCAGTTGATGAAAGTAC/<br>AT*GCTAGC*GATACATACATATTTATTTTCGAG (primes in 3'UTR)<br>deletion (fusion PCR):<br>GCTCACAATATTTTAAAACAAATACAGTAAATTGCTATG/<br>CTGTATTTGTTTTAAAATATTGTGAGCTGTTTTCTCTGC	pL0017-GFP-N; XbaI (compatible ends to NheI)
Pb^{con}PbMAPK2-GFP	pL0017-MCS1-PbMAPK2	EF1α	88	amplification of fragment:<br>AT*GCGGCCGC*ATGTTGAAAAAAAAAAAAGAATACTCTGG/<br>AT*GGATCC*ACTGCTAGGTTTCTCATTGTTATAGAAATTG	pL0017-MCS1; NotI/BamHI
Pb^{con}GFP-PbMAPK2	pL00017-GFP-N-PbMAPK2	EF1α	88	amplification of fragment:<br>AT*TCTAGA*ATGTTGAAAAAAAAAAAAGAATACTCTGG/<br>AT*TCTAGA*TTAACTGCTAGGTTTCTCATTGTTATAG	pL0017-GFP-N; XbaI
