# Supplementary material for: Plasmodium berghei MAPK1 Displays Differential and Dynamic Subcellular Localizations during Liver Stage Development
Source: PLoS One. 2013 Mar 27;8(3):e59755. doi: 10.1371/journal.pone.0059755 (PMC3609774; doi:10.1371/journal.pone.0059755)
Supplement: Table S2 — Vectors for expression of GFP-tagged PbMAPK1-CTD deletion constructs in HepG2 cells. (PDF) [file pone.0059755.s009.pdf]

Supplementary Table ST2: Vectors for expression of GFP-tagged PbMAPK1-CTD deletion constructs in HepG2 cells

vector	fusion protein	calculated molecular weight	oligonucleotides used (5' → 3')
pEGFP-C2-PbMAPK1-CTD	GFP-PbMAPK1-CTD WT	58 kDa	amplification of fragment:<br>AT*CTCGAG*TATTACAATACCAGTTGATGAAAGTAC/<br>AT*GGATCC*GATACATACATATTTATTTTCGAG (primes in 3'UTR)
pEGFP-C2-PbMAPK1-CTD-ΔNLS1	GFP-PbMAPK1-CTD- ΔNLS1	58 kDa	amplification of fragment:<br>AT*CTCGAG*TATTACAATACCAGTTGATGAAAGTAC/<br>AT*GGATCC*GATACATACATATTTATTTTCGAG (primes in 3'UTR)<br><br>site-directed mutagenesis (ΔNLS1):<br>CGAAATATTATATATTACAATATTTTAATTTTTATTCACAGAGAGCATTGCCC/<br>GGGCAATGCTCTCTGTGAATAAAAATTAAAATATTGTAATATATAATATTTCG
pEGFP-C2-PbMAPK1-CTD-ΔNLS2	GFP-PbMAPK1-CTD- ΔNLS2	57 kDa	amplification of fragment:<br>AT*CTCGAG*TATTACAATACCAGTTGATGAAAGTAC/<br>AT*GGATCC*GATACATACATATTTATTTTCGAG (primes in 3'UTR)<br><br>site-directed mutagenesis (ΔNLS2):<br>AATTATGATATGCATCAAATTGATTCACATTTTATATCTAATTCCCCAAATACACC/<br>GGTGTATTTGGGGAATTAGATATAAAATGTGAATCAATTTGATGCATATCATAATT

pEGFP-C2-PbMAPK1-CTD-ΔNLS1,2	GFP-PbMAPK1-CTD- ΔNLS1,2	57 kDa	amplification of fragment:<br>AT*CTCGAG*TATTACAATACCAGTTGATGAAAGTAC/<br>AT*GGATCC*GATACATACATATTTATTTTCGAG (primes in 3'UTR)<br>site-directed mutagenesis (ΔNLS1):<br>CGAAATATTATATATTACAATATTTTAATTTTTATTCACAGAGAGCATTGCCC/<br>GGGCAATGCTCTCTGTGAATAAAAATTAAAATATTGTAATATATAATATTTCG<br>site-directed mutagenesis (ΔNLS2):<br>AATTATGATATGCATCAAATTGATTCACATTTTATATCTAATTCCCCAAATACACC/<br>GGTGTATTTGGGGAATTAGATATAAAATGTGAATCAATTTGATGCATATCATAATT
pEGFP-C2-PbMAPK1-CTD-Δ1	GFP-PbMAPK1-CTD-Δ1	55 kDa	amplification of fragment:<br>AT*CTCGAG*TATTCACAGAGAGCATTGCCCAG/<br>AT*GGATCC*GATACATACATATTTATTTTCGAG (primes in 3'UTR)
pEGFP-C2-PbMAPK1-CTD-Δ2	GFP-PbMAPK1-CTD-Δ2	46 kDa	amplification of fragment:<br>AT*CTCGAG*ATCTAATTCCCCAAATACACCAAATGC/<br>AT*GGATCC*GATACATACATATTTATTTTCGAG (primes in 3'UTR)
pEGFP-C2-PbMAPK1-CTD-Δcc	GFP-PbMAPK1-CTD-Δcc	54 kDa	amplification of fragment:<br>AT*CTCGAG*TATTACAATACCAGTTGATGAAAGTAC/<br>AT*GGATCC*GATACATACATATTTATTTTCGAG (primes in 3'UTR)<br><br>deletion (fusion PCR):<br>GCTCACAATATTTTAAAACAAATACAGTAAATTGCTATG/<br>CTGTATTTGTTTTAAAATATTGTGAGCTGTTTTCTCTGC

pEGFP-C2-NLS1	GFP-MAPK1(NLS1)	29 kDa	oligonucleotide-annealing:<br>TCGAGTATTTTAAAAAGGAAAAAAATTTTTTGAG/<br>GATCCTCAAAAAATTTTTTTCCTTTTTAAAATAC
pEGFP-C2-NLS2	GFP-MAPK1(NLS2)	29 kDa	oligonucleotide-annealing:<br>TCGAGTGATTCAAAAAAAAAAACGAGGAAAAAAAAAAACGACATTTTTGAG/<br>GATCCTCAAAAATGTCGTTTTTTTTTTTCCTCGTTTTTTTTTTTGAATCAC
